# Pan-cancer stratification of solid human epithelial tumors and cancer cell lines reveals commonalities and tissue-specific features of the CpG island methylator phenotype

**DOI:** 10.1186/s13072-015-0007-7

**Published:** 2015-04-17

**Authors:** Francisco Sánchez-Vega, Valer Gotea, Gennady Margolin, Laura Elnitski

**Affiliations:** Translational and Functional Genomics Branch, National Human Genome Research Institute, National Institutes of Health, Bethesda, MD USA

**Keywords:** Cancer, DNA methylation, CpG island methylator phenotype, CIMP, TCGA, ENCODE, Pan-cancer

## Abstract

**Background:**

The term CpG island methylator phenotype (CIMP) has been used to describe widespread DNA hypermethylation at CpG-rich genomic regions affecting clinically distinct subsets of cancer patients. Even though there have been numerous studies of CIMP in individual cancer types, a uniform analysis across tissues is still lacking.

**Results:**

We analyze genome-wide patterns of CpG island hypermethylation in 5,253 solid epithelial tumors from 15 cancer types from TCGA and 23 cancer cell lines from ENCODE. We identify differentially methylated loci that define CIMP+ and CIMP− samples, and we use unsupervised clustering to provide a robust molecular stratification of tumor methylomes for 12 cancer types and all cancer cell lines. With a minimal set of 89 discriminative loci, we demonstrate accurate pan-cancer separation of the 12 CIMP+/− subpopulations, based on their average levels of methylation. Tumor samples in different CIMP subclasses show distinctive correlations with gene expression profiles and recurrence of somatic mutations, copy number variations, and epigenetic silencing. Enrichment analyses indicate shared canonical pathways and upstream regulators for CIMP-targeted regions across cancer types. Furthermore, genomic alterations showing consistent associations with CIMP+/− status include genes involved in DNA repair, chromatin remodeling genes, and several histone methyltransferases. Associations of CIMP status with specific clinical features, including overall survival in several cancer types, highlight the importance of the CIMP+/− designation for individual tumor evaluation and personalized medicine.

**Conclusions:**

We present a comprehensive computational study of CIMP that reveals pan-cancer commonalities and tissue-specific differences underlying concurrent hypermethylation of CpG islands across tumors. Our stratification of solid tumors and cancer cell lines based on CIMP status is data-driven and agnostic to tumor type by design, which protects against known biases that have hindered classic methods previously used to define CIMP. The results that we provide can be used to refine existing molecular subtypes of cancer into more homogeneously behaving subgroups, potentially leading to more uniform responses in clinical trials.

**Electronic supplementary material:**

The online version of this article (doi:10.1186/s13072-015-0007-7) contains supplementary material, which is available to authorized users.

## Background

DNA methylation plays an important role for cell fate commitment, both in disease and normal development [1-3]. Recurrent patterns of aberrant DNA methylation are commonly observed in cancerous cells, implying that this epigenetic alteration is inherently linked to general mechanisms of oncogenesis and tumor progression [4-7]. Since methylation of specific genomic loci is a potentially actionable event, the analysis of these patterns may influence therapeutic approaches aimed at individual subtypes of tumors [8-10]. Concurrent and widespread hypermethylation of CpG islands in clinically distinct cancer subtypes is known as *CpG island methylator phenotype* (CIMP) [11,12].

The concept of CIMP was introduced more than 15 years ago within the context of colorectal cancer [13], the cancer type for which it has been most extensively studied [14-17]. Since then, CIMP occurrence has been reported in a wide variety of additional tumor types (for a review, see Hughes *et al*. [11]). However, evidence for a pan-cancer overlap of individual gene targets is virtually absent in these previous reports, suggesting a tissue-specific CIMP program for each type of cancer [18].

In line with this, a number of genes have been implicated in CIMP outcomes in a tissue-specific manner. For example, the inactivation of mismatch repair gene *MLH1* [13] correlates strongly with CIMP in colon cancer. Glioblastoma exhibits mutations in epigenetic regulators such as *IDH1/2* and in histone encoding genes such as *H3F3A*, whereas CIMP in leukemia is associated with *TET2* mutations (for a review, see Witte *et al.* [19]). Despite these tissue-specific differences at the level of individual genes, there is a growing body of evidence that shows increased methylation targeting certain groups of genes within some cancer types [11,19,20]. The methylation targets are reproducible, not random, and the actual gene subgroups are strongly associated with specific molecular and pathological features, which reinforces the targeted nature of these events. More compelling evidence points to shared similarities in pathway analyses across tumors [21,22]. For example, targets of polycomb repressor complex (PRC) are frequently identified within hypermethylated gene sets and often involve tissue-specific developmental transcription factors [23]. However, to date, no consistently methylated targets have been identified across tumor types to represent a generalizable CIMP phenotype [19] and the question of whether or not CIMP is a universal phenomenon across cancers remains unclear [11].

We present a novel approach to stratify tumors based on molecular signatures of CIMP that are evaluated in a unified manner across different cancer types. Our proposed stratification can be used to refine current molecular subtyping, with important implications in terms of translation to the clinic. We also show that methylation levels averaged across a selected set of 89 CpG dinucleotides provide enough information to accurately distinguish CIMP+ tumors from CIMP− tumors across cancer types. This suggests that these loci are consistently targeted in CIMP across tissues and that average levels of methylation correlate to CIMP+ status. We demonstrate numerous statistically significant associations between CIMP status, genomic functional events, and clinical annotations that recapitulate several previously known results from the literature and therefore provide a means of *de facto* validation that supports the adequacy of our data-driven set of CIMP labels for patient stratification. Our analysis also gives rise to new biologically plausible hypotheses to be explored in future follow-up studies.

## Results

We analyzed DNA methylation data from the Illumina HumanMethylation450K platform for 5,253 solid tumors from 15 different cancer types made available by The Cancer Genome Atlas (TCGA) and for 51 cultured cell lines with known cancer (*n* = 23) or non-cancer (*n* = 28) origins made available by The Encyclopedia of Coding Elements (ENCODE). Based on reports of heterogeneous DNA methylation levels across a majority of tumor subtypes in recent studies [24-28], we hypothesize that the CIMP designation extends to subpopulations within the majority of cancer types and, therefore, that large cohorts of cancer patients may reveal a mixture of *CpG island methylator phenotype positive* (CIMP+) and *CpG island methylator phenotype negative* (CIMP−) tumor genomes.

### CIMP stratification of solid tumors and human cell lines

For each TCGA cancer type, we examined all probe locations within CpG islands (CGIs) with variable levels of DNA methylation by excluding probes with very low methylation variance (SD < 0.1, based on normalized beta values between 0 and 1). Probes located in chromosomes X and Y were also excluded from these sets. To assess the extent of aberrant hypermethylation in different types of cancer, we first analyzed genome-wide variation of CGI methylation in tumors *vs.* controls (Additional file 1: Figure S1 and Additional file 2). These plots visually demonstrate the distribution of methylated and unmethylated probes, whereby a large fraction of the variably methylated sites have very low levels of methylation in controls and become aberrantly hypermethylated in tumors (Additional file 1: Figure S1A). Also, a vast majority of these sites exhibit larger standard deviation in methylation levels across tumor samples than across controls (Additional file 1: Figure S1B), which is consistent with previous reports of increased methylation variability in cancer [29]. The number of variably methylated probes for each cancer type varied from 21,945 to 62,606 out of the 485,512 probes in the array (Table 1). 

We examined each cancer type separately and we focused our analysis on patterns of differential methylation occurring at sites of minimal methylation in control samples and increased methylation in tumors. For this, we selected probes with average methylation levels below 5% across controls and average methylation levels above 25% across tumors. This dual-thresholding approach and the actual choice of thresholds guaranteed a very small probability of spurious detections for probe selection, as we showed using Monte Carlo simulations and random permutations of probe labels and beta-values (see the ‘Methods’ section). The number of differentially methylated probes selected for each cancer type ranged from 0 to 2,656 (Table 1, the actual sets of differentially methylated probes selected for each individual cancer type are available as Additional file 3). Cases like uterine corpus endometrioid carcinoma (UCEC) and colon adenocarcinoma (COAD), with a known CIMP phenotype [13,24-26], had 1,430 and 2,656 differentially methylated probes, respectively. Thyroid carcinoma (THCA) was the only type for which no probes were selected, likely indicating no methylator phenotype within the actual samples in the data set that we used, so we excluded it from the rest of the study.

**Table 1:** **Cancer types, sample sizes and probe-set cardinalities**

**Cancer type**	**Variably methylated probes**	**Differentially methylated probes**	**Control**	**Tumor**	**CIMP-**	**CIMPi**	**CIMP+**	**CIMP−**	**CIMP+**
								**pan-cancer**	**pan-cancer**
BLCA (bladder urothelial carcinoma)	49,148	338	20	201	78	84	39	43	14
BRCA (breast invasive carcinoma)	46,722	1,311	96	676	270	244	162	76	47
COAD (colon adenocarcinoma)	46,168	2,656	38	274	96	92	86	71	60
HNSC (head and neck squamous cell carcinoma)	44,100	1,228	50	426	156	186	84	115	55
KIRC (kidney renal clear cell carcinoma)	26,148	196	160	296	126	94	76	97	65
KIRP (kidney renal papillary cell carcinoma)	28,083	40	45	147	60	59	28	NA	NA
LIHC (liver hepatocellular carcinoma)	51,875	544	50	151	45	61	45	NA	NA
LUAD (lung adenocarcinoma)	42,822	1,667	32	437	161	169	107	67	48
LUSC (lung squamous cell carcinoma)	40,606	1,430	42	359	140	142	77	32	11
PAAD (pancreatic adenocarcinoma)	27,899	1,602	9	65	16	33	16	NA	NA
PRAD (prostate adenocarcinoma)	33,718	450	49	248	74	122	52	NA	NA
READ (rectum adenocarcinoma)	40,496	1,255	7	96	31	39	26	22	22
STAD (stomach adenocarcinoma)	62,606	1,110	2	260	109	95	56	NA	NA
THCA (thyroid carcinoma)	21,945	0	56	508	NA	NA	NA	NA	NA
UCEC (uterine corpus endometrioid carcinoma)	43,040	1,430	46	407	155	139	113	54	34

Probe set cardinalities and sample sizes for the 15 cancer types that were included in our analysis. The last two columns show the number of CIMP+ and CIMP− samples from our genome-wide methylation study that also appear in the selected functional event data matrix from Ciriello *et al*. [27].

#### Classification of tumor samples into CIMP subtypes

**Figure 1:**
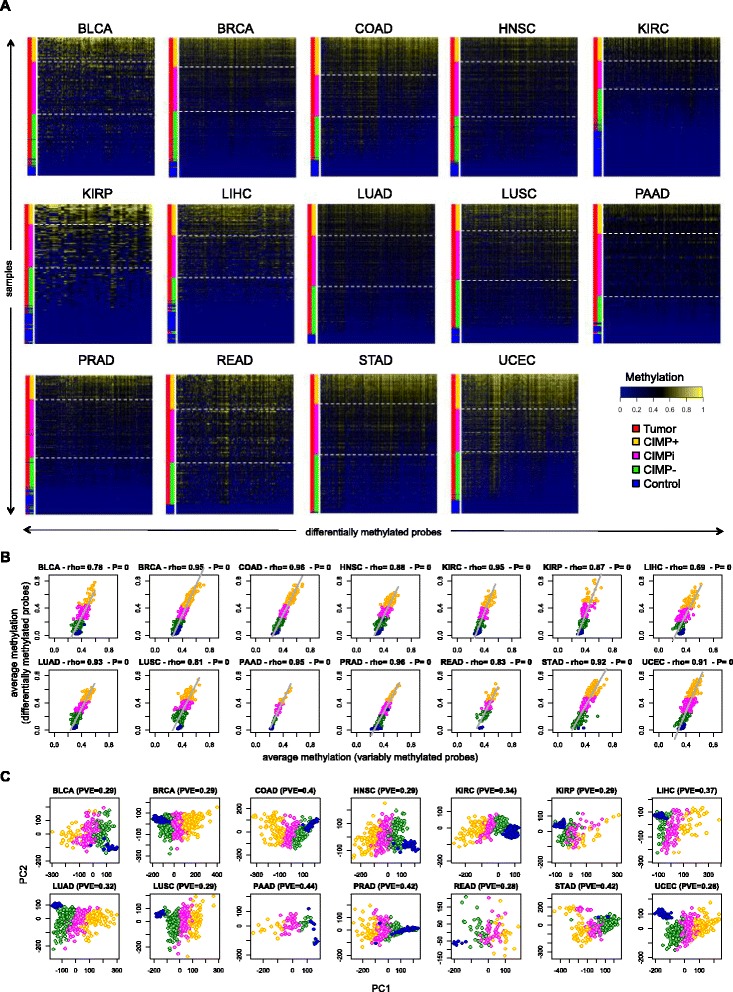
CIMP+ and CIMP− samples across cancer types. **(A)** Heat maps showing differentially methylated probes for each individual cancer type. Rows and columns represent samples and selected probes, respectively. Color side bars show tumor *vs*. control labels, as well as CIMP+, CIMPi, and CIMP− labels resulting from k-means clustering on the vector of average methylation values computed over differentially methylated sites. Rows were ranked from top to bottom in decreasing order of average methylation computed over selected probes. Columns were ordered horizontally using hierarchical correlational clustering. White dashed horizontal lines were used to highlight different subgroups based on CIMP status. **(B)** Average sample methylation computed over the sets of variably methylated probes (horizontal axes) *vs*. average sample methylation computed over the set of selected differentially methylated probes (vertical axes). For each plot, we provide the Spearman rho coefficient and the corresponding *P*-value. **(C)** PCA results where samples are projected onto the first two principal components. PCA was computed using data for all variably methylated probes within each cancer type. For each plot, we provide the corresponding percentage of variance explained (PVE) by the first two principal components. In panels **(B)** and **(C)**, each point represents an individual sample and samples are colored according to their CIMP status, using the same color labels as in **(A)**. THCA was excluded from the three panels because no differentially methylated probes had been selected for it.

We stratified samples into groups that are representative of CIMP status by classifying all tumors within each cancer type into three different categories using k-means clustering of mean methylation values computed over the tumor-specific probe sets (Figure 1A and Additional file 2). We labeled tumor samples with the lowest average levels of methylation as *CIMP−* and those with the highest average levels of methylation as *CIMP+*. We noted that clustered heat maps of the data show a gradient of DNA methylation levels across the probe sets from CIMP− to CIMP+. For the purposes of our subsequent computational and functional analysis, we focused on these two sample categories and excluded tumors assigned to the intermediate group (that we refer to as *CIMPi*). Eliminating samples classified as CIMPi allows unambiguous classification of tumors with strong biological differences that are most representative of CIMP extremes, at the price of a reduced effective sample size for statistical comparisons. We assessed the robustness of our sample stratification across a wide range of probe selection thresholds and found that the actual choice of cutoff values did not change our assignment of CIMP+/− labels in a relevant manner (see Additional file 2). Also, the CIMP+/− groups remained largely unaltered when probes were chosen using an alternative strategy based on variance-guided feature selection (Additional file 2). The number of control, tumor, CIMP+, CIMP−, and CIMPi samples for each cancer type is provided in Table 1 (individual sample labels are available as Additional file 4).

We questioned whether aberrant methylation represents a more widespread alteration of the genomic landscape by exploring correlations between the differentially methylated probe sets selected for each tissue type and the much larger sets of variably methylated probes in CpG islands (Figure 1B). These probe sets differ in size by an order of magnitude (Table 1). The strong correlation indicated that average levels of methylation measured over the relatively small sets of differentially methylated probes recapitulated the same levels measured over the larger, more extensive sets involving tens of thousands of CpG dinucleotides located in CpG islands across the genome. This conclusion indicates that a wide-spread aberrant methylation process occurs and that it can be modeled within each individual cancer type by a distinct set of differentially methylated probes that exhibit a large and consistent magnitude of effect. This finding was further supported by principal component analysis (PCA) plots computed over the large sets of variably methylated probes, where our CIMP classification labels were always grouped in spatially coherent clusters with the CIMPi tumors separating the CIMP+ from the CIMP− group (Figure 1C).

**Figure 2:**
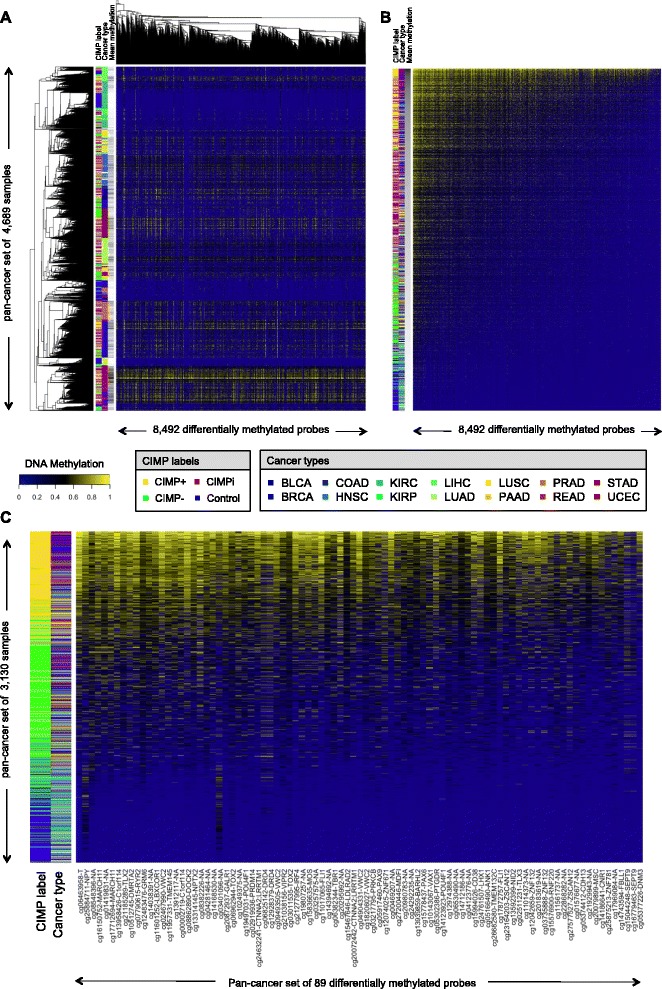
Pan-cancer clustering of TCGA tumors based on DNA methylation levels. Heat maps show levels of DNA methylation for TCGA tumor and control samples. Samples were pooled together across 14 cancer types (all except THCA). Each row corresponds to a sample and each column corresponds to a probe. Color bars show the CIMP status and the cancer type associated to each sample. **(A)** Heat map showing results for CIMP+, CIMPi, CIMP−, and control samples over a reference pan-cancer set of 8,492 probes (obtained as the union of type-specific sets of differentially methylated probes). Rows and columns were ordered using hierarchical correlational clustering. **(B)** Same as panel (A), but rows and columns were ranked in decreasing order of average methylation, from top to bottom and from left to right, respectively. **(C)** Same as panel (B), but average levels of methylation were computed using our proposed panel of 89 pan-cancer differentially methylated loci. In panels **(A)** and **(B)**, a third color bar shows the relative ranking of each sample in terms of average methylation, with black showing the most methylated sample and white showing the least methylated sample. In panel **(C)**, CIMPi tumors were excluded to facilitate visual comparison of the CIMP+/− categories and, for probes associated to known genes, the actual gene or genes are included next to each probe identifier.

In order to further investigate similarities in CIMP across different cancer types, we drew pan-cancer heat maps of the entire set of samples, pooling together tumors and controls from different cancer and tissue types (Figure 2). We considered data from a reference set of 8,492 probes representing the union of our tissue-specific sets of differentially methylated loci. When we looked at hierarchical correlational clustering of beta values, we observed that tumors clustered according to cancer type (Figure 2A), which was consistent with previous reports of tissue-of-origin largely characterizing DNA methylation patterns in tumor cells [21]. In contrast, when we ranked samples according to their average levels of methylation over the same set of 8,492 probes, we observed that tumors clustered according to our definitions of CIMP status rather than according to cancer type (Figure 2B).

#### Identification of a pan-cancer panel of CIMP markers

After selecting tumor type-specific differential probe sets for individual cancer types, we searched for discriminative loci that were consistently chosen across multiple cancer types. We identified a minimal set of 89 differential probes that was present in at least 6 of 14 selected probe sets from different cancer types (Additional file 5: Table S1). This threshold of 6/14 was chosen as a trade-off between presence in as many cancer types as possible and the need to select a sufficiently large number of probes. We performed a leave-one-type-out (LOTO) cross-validation analysis in order to assess the ability of this set of markers to separate CIMP+ from CIMP− samples. Based on Monte Carlo simulation, the classification rates and correlations with genome-wide levels of CGI methylation were statistically significant for all cancer types except kidney renal papillary cell carcinoma (KIRP) and stomach adenocarcinoma (STAD), which were consequently excluded from the rest of our study (Additional file 2). The pan-cancer panel of 89 selected loci achieved a classification accuracy of 97.57%, averaged over the 12 cancer types with statistically significant classification rates (Additional file 5: Table S2).

A pan-cancer ranking of samples based on average levels of methylation computed over our proposed panel of 89 markers corroborates that this set can distinguish CIMP+ from CIMP− samples with very high accuracy (Figure 2C). Together with the pan-cancer heat maps presented earlier, these results illustrate that CIMP+ tumors show consistent elevation in average CGI methylation levels among multiple cancer types (Figure 2B,C), even though an important fraction of this hypermethylation is distributed in tissue-specific patterns (Figure 2A). Additionally, CIMP− tumors tend to have lower average methylation levels, although these are still higher than baseline non-cancer controls. The consistent behavior of the small pan-cancer panel of 89 loci across the evaluated cancer types demonstrates its efficacy in cross-cancer determination of CIMP status.

#### Canonical pathways and upstream regulators

**Figure 3:**
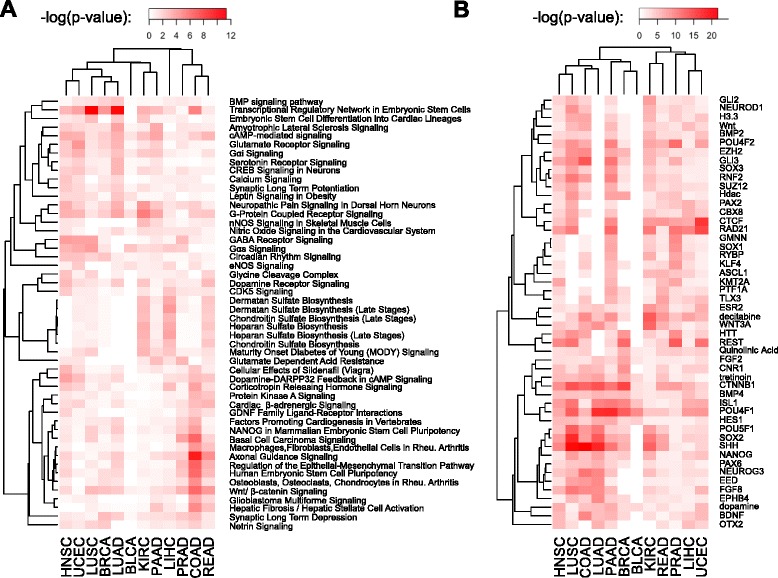
Canonical pathways and upstream regulators associated to selected differentially methylated sites across cancer types. **(A)** Enrichment of canonical pathways associated to genes that are interrogated by selected differentially methylated probes. **(B)** Enriched upstream regulators of selected probes. Heat map colors show –log(*P*-values), so that more intense red color corresponds to higher statistical significance. Each panel shows the top 50 scorers based on Fisher’s sum for combining *P*-values. Rows correspond to pathways or regulators, while columns correspond to different cancer types. Rows and columns were ordered using hierarchical correlational clustering.

An Ingenuity Pathway Analysis (IPA) evaluation of the genes associated with the differential methylation sites in individual cancers revealed a subset of canonical pathways that are collectively targeted in the CIMP probe sets (Figure 3A). Several of these were related to tissue morphology and development, including regulation of pluripotency in embryonic stem cells. We also observed an important enrichment of genes involved in the Wnt/beta-catenin pathway, pathways involved in glutamate receptor signaling, and regulators of epithelial-mesenchymal transition (EMT). These pathway enrichments create potentially interesting clusters among the different tumor types, forming subgroups for head and neck squamous cell carcinoma (HNSC), UCEC, lung squamous cell carcinoma (LUSC), breast invasive carcinoma (BRCA), and lung adenocarcinoma (LUAD); kidney renal clear cell carcinoma (KIRC) and pancreatic adenocarcinoma (PAAD); and prostate adenocarcinoma (PRAD), rectum adenocarcinoma (READ), and COAD. Using the IPA tool, we also identified a set of recurrent upstream regulators for the differentially methylated probe set associated with each cancer type (Figure 3B). These included important members of the PRC (*SUZ12*, *EZH2*), chromatin remodeling genes (*CTCF*, *HDAC*), histone coding genes (*H3F3A*), members of the Wnt/beta-catenin pathway (*WNT*, *CTNNB1*), genes known to be important for embryonic stem cell differentiation (*NANOG*, *SOX2*, *POU5F1/OCT4*), and several members of the sonic hedgehog pathway (*SHH*, *OTX2*, *PAX6*, *GLI3*).

#### Assessment of CIMP status in ENCODE cell lines

**Figure 4:**
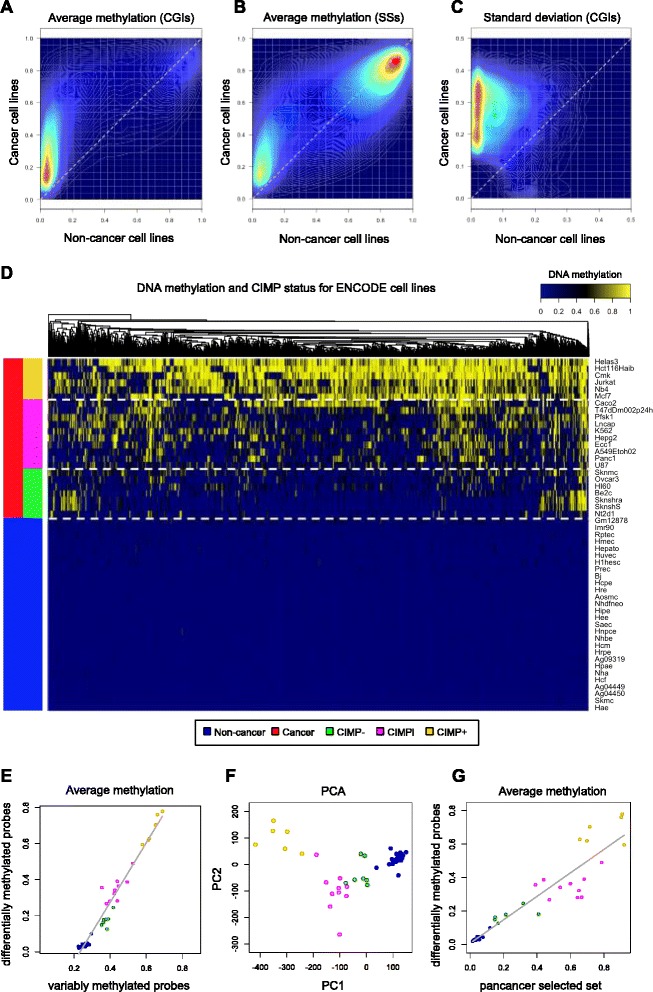
Characterization of CIMP in ENCODE cell lines. **(A)** Density plot of average site methylation for variably methylated in-CGI probes in cancer *vs*. non-cancer cell lines. **(B)** Same plot for probes in CGI shores and shelves. **(C)** Density plot showing standard deviation for variably methylated sites. **(D)** Heat map showing results from the CIMP classification algorithm. **(E)** Average cell line methylation computed over selected differentially methylated probes *vs*. average methylation computed over variably methylated probes. **(F)** PCA results showing samples projected onto the first two principal components and colored according to their CIMP status. **(G)** Average cell line methylation computed over variably methylated probes (vertical axis) *vs*. average methylation computed over set of 89 pan-cancer selected differentially methylated probes (horizontal axis).

We applied our computational pipeline for CIMP identification to methylation data from a heterogeneous panel of 51 ENCODE cell lines. We evaluated all cell lines from the ENCODE HAIB track at the UCSC Genome Browser that had a cancer or non-cancer origin (*n* = 23 and *n* = 28, respectively), based on available ENCODE annotations (Additional file 5: Table S3). The set of variably methylated probes included 69,186 loci. The overall methylation patterns at these sites in cancer *vs*. non-cancer cell lines resembled our observed patterns for solid epithelial tumors compared to normal samples (Figure 4A,B,C). Our feature selection algorithm chose a set of 8,702 differentially methylated probes. K-means classification of average methylation values computed over that set identified 6, 10, and 7 cell lines as CIMP+, CIMPi, and CIMP−, respectively (Figure 4D). CIMP+ samples included HeLa and Jurkat cell lines, whereas CIMP− samples included HL60 and Nt2d1. Non-tumor cell lines were treated as controls in the feature selection algorithm, and therefore, they show baseline methylation levels at all selected probe sites. As we had observed in solid epithelial tumors, the reduced set of differentially methylated probes were highly correlated with genome-wide variably methylated probes (Figure 4E) and our CIMP labels revealed coherent clusters on a PCA plot (Figure 4F). Also, we noted a strong correlation between the differentially methylated probe set and the set of 89 tumor-derived pan-cancer loci (Figure 4G), which supports the consistency of our findings between the cell lines and the tumor data. In fact, a majority of the 89 pan-cancer probes (80%, 71 of 89) were included in the cell line specific, differentially methylated probe set.

### Pan-cancer neighborhoods of hyper- and hypomethylation

In assessing pan-cancer patterns of aberrant methylation, we searched for consecutive probes that show consistent patterns of change. This allowed us to reduce the number of effective candidate regions that needed to be evaluated in our subsequent analysis. Using an unsupervised clustering algorithm, we identified probes with similar levels of differential DNA methylation in the CIMP+ and CIMP− samples across multiple tissues (see Additional file 2). Starting from the original set of 485,512 probes in the Illumina array, this approach identified a total of 105,875 clusters of probes that were differentially methylated at statistically significant levels for at least one of the 12 cancer types under consideration (Additional file 5: Table S4). About two thirds of the regions (66%, 69,946 of 105,875) were associated with known genes (that is, fell within 1.5 kb of the annotated transcripts).

**Figure 5:**
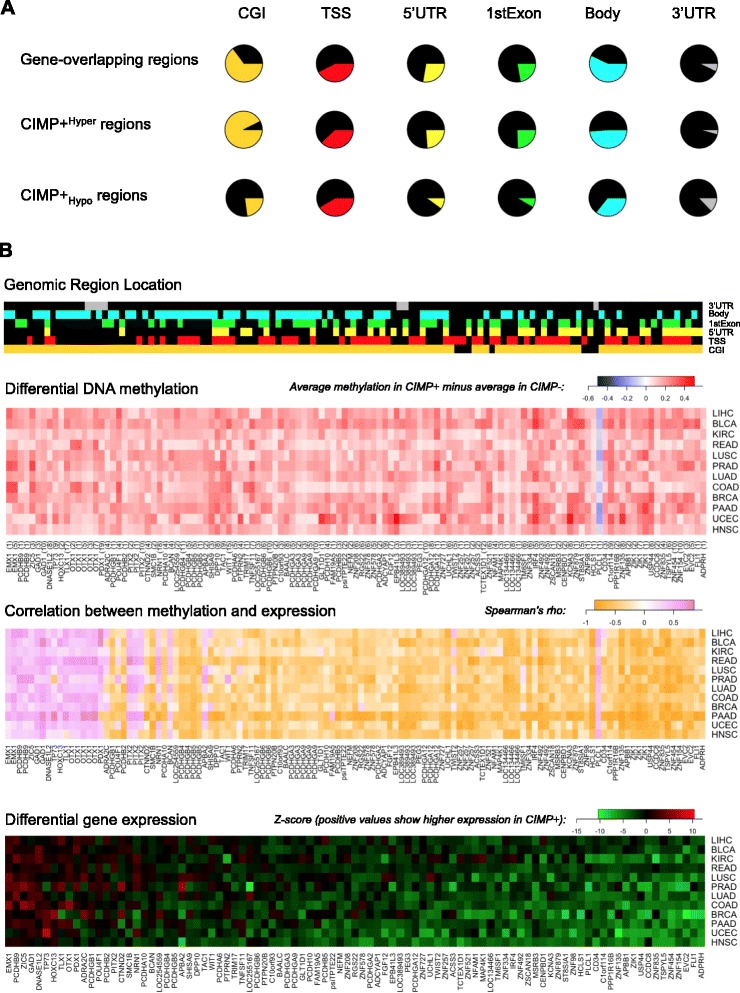
Differentially methylated regions and differentially expressed genes in CIMP+ relative to CIMP- samples from TCGA. **(A)** Proportion of gene-associated regions, CIMP + ^Hyper^ regions and CIMP + _Hypo_ regions overlapping CGIs, TSSs, 5′ UTRs, first exons, gene bodies, and 3′ UTRs. **(B)** Differentially expressed genes exhibiting significant correlation with methylation at associated CIMP + ^Hyper^ or CIMP + _Hypo_ regions. The 93 genes selected in the bottom panel overlapped at least one CIMP + ^Hyper^ or one CIMP + _Hypo_ region and exhibited significant levels of Spearman correlation (FDR < 0.10) in all the 12 cancer types that we analyzed. Top color bars shows genomic locations of probes within each of the 120 CIMP + ^Hyper^ and 1 CIMP + _Hypo_ regions overlapping one of those 93 genes. Top heat map shows differences in mean methylation for these 121 regions. Middle heat map shows values of Spearman correlation between methylation within these 121 regions and expression of the 93 associated genes. Bottom panel shows differential expression (Z-scores) for these 93 genes in CIMP+ *vs*. CIMP− samples, with red corresponding to genes with higher expression levels in CIMP+. Rows and columns in the bottom heat map were ordered according to average Z-score, decreasing from left to right and from top to bottom. Columns in the middle and top heat map were drawn so that genes associated to differentially methylated regions were shown in the same order as in the bottom heat map. Row order was also chosen to be the same as in the bottom heat map. The number of array probes located within each CIMP + ^Hyper^ or CIMP + _Hypo_ region is shown in parentheses after the corresponding gene name below the differential methylation heat map.

We divided the dataset into regions of pan-cancer hypermethylation in CIMP+ samples (*CIMP +* ^*Hyper*^*regions*) by requiring the mean level of methylation in CIMP+ samples (averaged over all the probes within the region) to be at least 5% higher than in CIMP− samples (that is, average differences of at least 0.05 in beta values) and that this minimum difference be observed for all 12 cancer types. We defined *CIMP +*_*Hypo*_*regions* in an analogous manner, but requiring that average levels of methylation in CIMP+ samples be at least 5% lower than in CIMP− samples. The 5% minimum difference was chosen to enforce non-negligible magnitude of effect (on top of the statistically significant differences used to define the clusters) and acted as a strong requirement when imposed simultaneously upon all the 12 cancer types. We identified 6,408 *CIMP +* ^*Hyper*^ regions and 68 CIMP + _Hypo_ regions. A total of 3,892 *CIMP +* ^*Hyper*^ regions were associated with at least one gene, covering 1,805 distinct genes. A total of 54 distinct genes were associated with 54 distinct CIMP + _Hypo_ regions. We identified *CIMP +*_*Hypo*_ and *CIMP +* ^*Hyper*^ regions appearing concurrently within 18 genes, including five zinc-finger genes (*RNF144A*, *ZNF727*, *ZNF536*, *ZIK1*, and *ZSCAN1).* These discordant alterations strongly implicate disruption of the normal regulation of these genes. Consistent with our prior findings, an IPA analysis of canonical pathways and upstream regulators using these differentially methylated regions yielded results that largely coincided with the ones that we had reported using differentially methylated probes (as was shown in Figure 3). Of note, the *CIMP +* ^*Hyper*^ region with the largest minimum increase in average methylation in every single cancer type (≥30%) was associated with the T-box transcription factor Brachyury, which has been reported to drive primary tumors towards metastasis under certain conditions by inducing EMT [30]. Of note, the probe in the pan-cancer set of 89 loci that exhibited the largest magnitude of effect in terms of average hypermethylation across samples was also associated to this gene (Figure 2C). A vast majority of *CIMP +* ^*Hyper*^ regions overlapped CGIs, which was not the case for *CIMP +*_*Hypo*_ regions (Figure 5A). Furthermore, less than half of the *CIMP +* ^*Hyper*^ regions in the gene-associated set collocated with known transcription start sites annotated by Illumina (and a comparable number overlapped gene bodies), suggesting that aberrant hypermethylation in CIMP is not exclusive to gene promoters. However, we cannot rule out regions of unannotated alternative promoters or unannotated promoters of novel transcripts.

#### Identification of differentially methylated and differentially expressed genes

To test hypotheses that DNA methylation events affect gene regulatory programs, RNA-Seq data generated by TCGA were used to assess correlations between methylation and expression for genes overlapping with *CIMP +*_*Hypo*_ and *CIMP +* ^*Hyper*^ regions. A subset of 121 regions associated with 93 genes exhibited significant levels of Spearman correlation between methylation and expression in all 12 cancer types, with varying magnitudes of effect in terms of actual differential expression (Figure 5B). The gene with the strongest global difference in gene expression was *ADPRH*, which is involved in DNA repair through histone ADP-ribosylation [31] and has been shown to play a role in tumorigenesis in mice [32]. Additionally, *FLI1* (which had been identified in our selected differentially methylated probe sets for 8 of 14 cancer types), contained a combination of *CIMP +* ^*Hyper*^ and *CIMP +*_*Hypo*_ regions, which occurred at the gene promoter and the first exon, respectively. Only one *CIMP +*_*Hypo*_ region exhibited consistent levels of significant correlation with expression across all 12 cancer types, located at the 3′UTR of gene *PLCL1*. We also identified families of genes showing consistently significant correlations between expression and differential methylation, including 14 genes from the protocadherin family and 16 zinc-finger genes. In particular, *ZNF154* - which we recently proposed as a pan-cancer biomarker to distinguish tumors from non-cancer controls [28] - was also part of this collection.

### Analysis of associations between methylator phenotype and composite functional profiles

One hypothesis to explain the occurrence of CIMP across different tissues is that it arises due to some shared underlying genomic alterations or functional mechanisms. To address commonalities, we used characterizations of TCGA data generated by Ciriello *et al*. [27], which consisted of 479 selected functional events (SFEs) including 116 copy number gains, 151 copy number losses, mutation of 199 genes, and epigenetic silencing of 13 genes (requiring promoter methylation and decreased expression). Although not specifically reported as driver alterations, these events had originally been chosen due to their relevance in cancer. The study intersected 9 of the 15 TCGA tumor collections from our analysis: bladder urothelial carcinoma (BLCA), BRCA, COAD, HNSC, KIRC, LUAD, LUSC, READ, and UCEC.

**Figure 6:**
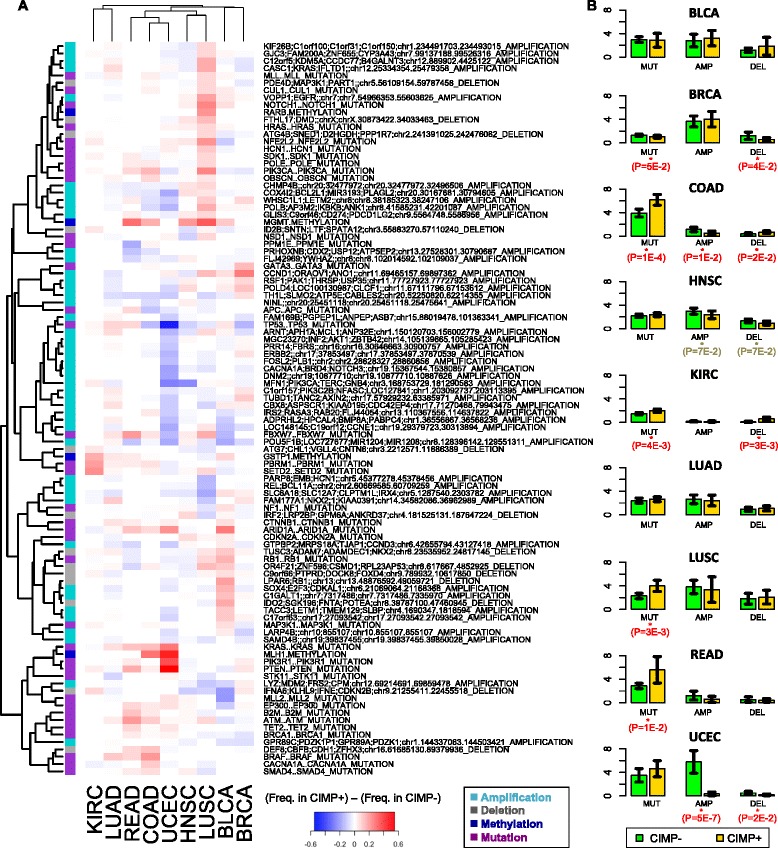
Frequency of SFE occurrence in CIMP+ *vs*. CIMP− samples from TCGA. **(A)** The heat map shows frequency of occurrence in CIMP+ samples minus frequency in CIMP- samples for the top 100 SFEs with the greatest absolute variation across cancer types. Each row corresponds to a SFE and each column corresponds to a different cancer type. The color side bar shows the category associated to each SFE (amplification, deletion, mutation or methylation event). **(B)** Average number of mutations, amplifications and deletions per sample in different types of cancer. Error bars show 95% confidence intervals. Reported *P*-values were computed using a one sided *t*-test.

For each cancer type, we compared the average frequencies associated with each functional event in CIMP+ samples and CIMP− samples. From a matrix of differential frequencies, we selected the top 100 functional events with the highest average absolute differences (Figure 6A). Events with strong effects in more than one tumor type included *MGMT* and *MLH1* promoter methylation, as well as mutation of *ARID1A*, *KRAS*, *BRAF*, and *PTEN*. Events that were strong but gave mixed results towards the CIMP phenotype included mutation of *TP53*, *PIK3CA*, *FBXW7*, and several amplification and deletion regions. We performed an aggregation analysis where we looked for differences in SFE frequencies between the entire set of pooled CIMP+ samples and the entire set of pooled CIMP− samples (including 356 CIMP+ samples and 577 CIMP− samples from all 9 cancer types combined) to test for the possibility of universal or convergent pathway events. The top 20 SFEs in terms of differences in frequencies are shown in Table 2, wherein a total of 12 SFEs showed statistically significant differences (FDR < 0.10). Of these, four amplification events were significantly more frequent in CIMP− samples and involved genes such as *PIK3CA*, *TERC*, *SOX2*, *CCNE1*, *BRD4*, and *NOTCH3*. In contrast, mutations in six genes (*BRAF*, *PTEN*, *KRAS*, *SETD2*, *PIK3R1*, and *PBRM1*) and two gene silencing events (*MLH1* and *MGMT*, for which the smallest FDRs were recorded) were more frequent in CIMP+ samples. Of interest within the context of CIMP, *PBRM1* is a SWI/SNF chromatin remodeling complex gene that has been reported to play a tumor suppressor role across multiple cancer types [33], and *SETD2* is a histone methyltransferase for H3K36 methylation, which is enriched in the gene bodies of actively transcribed genes [34]. The remaining mutations are well known due to their involvement in the *PI3K/PTEN/AKT/mTOR* [35] and the *Ras/Raf/MEK/ERK* [36] pathways. In particular, both *BRAF* [37] and *KRAS* [38,39] mutations have been linked to CIMP status (high and low, respectively) in colorectal cancer [37-39]. Despite these associations, the mechanistic connections to CIMP are not discernable from the reported functional activities of these proteins and the prominent driver mutations do not appear to be responsible for a ‘universal’ methylator phenotype.

**Table 2:** **Top 20 SFEs in terms of differential frequencies in pooled CIMP+** ***vs*** **. pooled CIMP− samples**

**Selected functional event**	**DifFreq**	**Count CIMP+**	**Freq CIMP+**	**Count CIMP−**	**Freq CIMP−**	***P*** **-value**	**FDR**
**MLH1.METHYLATION**	**0.100**	**38**	**0.107**	**4**	**0.01**	**7.11 × 10** ^**−13**^	**3.40 × 10** ^**−10**^
**MGMT.METHYLATION**	**0.113**	**58**	**0.163**	**29**	**0.05**	**2.22 × 10** ^**−08**^	**5.30 × 10** ^**−06**^
**MFN1.PIK3CA.TERC.GNB4.SOX2.MECOM.ZMAT3.KCNMB3.ZNF639.KCNMB2..chr3.168753729.181290583_AMP**	**−0.094**	**17**	**0.048**	**82**	**0.14**	**3.06 × 10** ^**−06**^	**4.89 × 10** ^**−04**^
**BRAF..BRAF_MUTATION**	**0.063**	**30**	**0.084**	**12**	**0.02**	**1.15 × 10** ^**−05**^	**1.38 × 10** ^**−03**^
**PTEN..PTEN_MUTATION**	**0.080**	**47**	**0.132**	**30**	**0.05**	**2.48 × 10** ^**−05**^	**2.38 × 10** ^**−03**^
**KRAS..KRAS_MUTATION**	**0.091**	**62**	**0.174**	**48**	**0.08**	**4.07 × 10** ^**−05**^	**3.25 × 10** ^**−03**^
**SETD2..SETD2_MUTATION**	**0.053**	**25**	**0.070**	**10**	**0.02**	**5.86 × 10** ^**−05**^	**4.01 × 10** ^**−03**^
**LOC148145.C19orf12.CCNE1..chr19.29379723.30313894_AMP**	**−0.044**	**3**	**0.008**	**30**	**0.05**	**2.02 × 10** ^**−04**^	**1.21 × 10** ^**−02**^
**CACNA1A.BRD4.NOTCH3..chr19.15367544.15380857_AMP**	**−0.035**	**1**	**0.003**	**22**	**0.04**	**2.98 × 10** ^**−04**^	**1.59 × 10** ^**−02**^
**PIK3R1..PIK3R1_MUTATION**	**0.046**	**25**	**0.070**	**14**	**0.02**	**1.10 × 10** ^**−03**^	**5.28 × 10** ^**−02**^
**DNM2..chr19.10877710.10887626_AMP**	**−0.024**	**0**	**0.000**	**14**	**0.02**	**1.46 × 10** ^**−03**^	**6.35 × 10** ^**−02**^
**PBRM1..PBRM1_MUTATION**	**0.058**	**43**	**0.121**	**36**	**0.06**	**2.37 × 10** ^**−03**^	**9.46 × 10** ^**−02**^
ATM..ATM_MUTATION	0.042	25	0.070	16	0.03	2.82 × 10^−03^	1.01 × 10^**−01**^
TP53..TP53_MUTATION	−0.101	143	0.402	290	0.50	2.94 × 10^−03^	1.01 × 10^**−01**^
ARID1A..ARID1A_MUTATION	0.052	40	0.112	35	0.06	6.16 × 10^−03^	1.95 × 10^**−01**^
GSTP1.METHYLATION	0.054	44	0.124	40	0.07	6.51 × 10^−03^	1.95 × 10^**−01**^
SAMD4B..chr19.39837455.39850028_AMP	−0.024	2	0.006	17	0.03	1.47 × 10^−02^	3.76 × 10^**−01**^
TMED11P.CTBP1.SPON2..chr4.1105313.1243877_DEL	−0.016	0	0.000	9	0.02	1.55 × 10^−02^	3.76 × 10^**−01**^
KEAP1..KEAP1_MUTATION	−0.016	0	0.000	9	0.02	1.55 × 10^−02^	3.76 × 10^**−01**^
FOSL2.PLB1..chr2.28628327.28860856_AMP	−0.017	0	0.000	10	0.02	1.65 × 10^−02^	3.76 × 10^**−01**^

Data included 356 CIMP+ samples and 577 CIMP− samples. *P*-values were computed using a two-sided Fisher’s exact test. SFEs with FDR < 0.10 are highlighted in bold font.

We compared the average counts of copy number events and mutational events per sample in the CIMP+ and the CIMP− subsets for each individual cancer type (Figure 6B). CIMP+ samples for COAD, KIRC, LUSC, and READ exhibited a larger number of mutational events per sample than CIMP− samples, implicating impairment of DNA repair processes. In contrast, copy number variation showed significant effects in CIMP− samples, where amplifications occurred more frequently in COAD and UCEC tumors, and deletions occurred more frequently in BRCA and UCEC tumors. However, these events are not always positively correlated, as shown by the reduction in deletions in COAD CIMP− samples.

**Figure 7:**
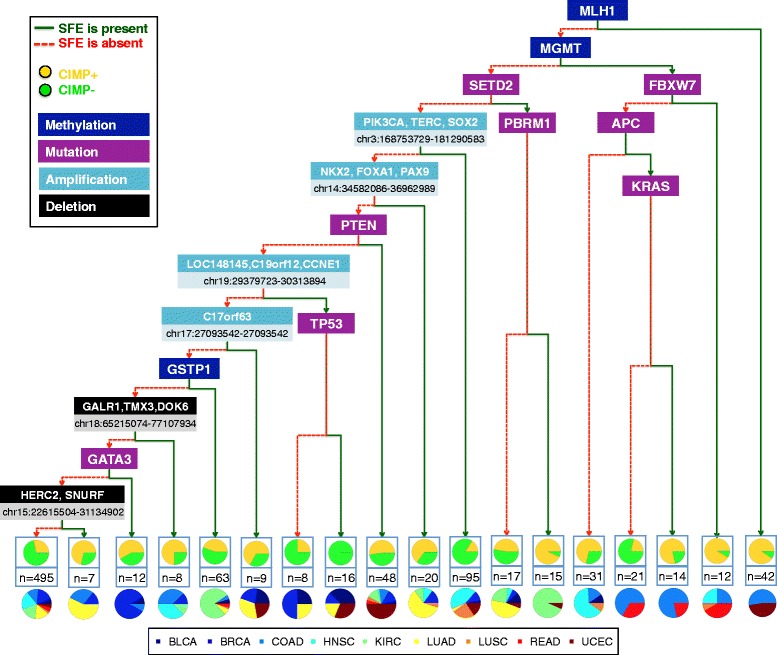
Pan-cancer partitioning of TCGA tumors using a binary classification tree. Pan-cancer binary tree for classification of tumor samples into the CIMP+ and the CIMP− categories. Red and green branches illustrate the absence or presence of the corresponding SFE, respectively. Terminal nodes show the number of samples and associated fractions of CIMP+ *vs*. CIMP− labels, as well as proportions of different cancer types represented in each subset.

Finally, we used binary classification and regression trees on these SFEs in order to identify individual features that were able to recursively partition the original set of tumors into increasingly homogeneous subgroups based on either CIMP status (in the classification case, Figure 7) or average methylation levels from the variably methylated probe sets (in the regression case, Additional file 1: Figure S2). We learned pan-cancer trees by pooling together samples across different cancer types (Figure 7 and Additional file 1: Figure S2), and we also learned type-specific trees on individual cancer types (Additional file 1: Figures S3 and S4). The pan-cancer trees highlight SFEs which are relevant in more than one type of tumor. For example, *MLH1* promoter methylation is observed in a subset of COAD and UCEC tumors with a very strong majority of CIMP+ labels. Similarly, a high proportion of CIMP+ labels was observed in samples with *MGMT* promoter methylation, combined with either (a) *FBXW7* mutations or (b) *APC* and *KRAS* mutations or (c) absence of *FBXW7* and *APC* mutations (Figure 7). Of note, subgroups containing these alterations consisted entirely of tumors of the aero-digestive tract (HNSC, LUSC, COAD, and READ). Notably, *MLH1* and *MGMT* have been previously associated with two distinct methylation landscapes in colorectal cancer that exhibited important differences in terms of *KRAS* and *APC* mutation frequency [40].

In our samples lacking *MGMT* and *MLH1* promoter methylation, the highest proportions of CIMP+ samples were observed in a subgroup dominated by KIRC tumors that were characterized by a combination of *SETD2* and *PBRM1* mutations. We found co-occurrence of *CCNE1* amplification and *TP53* mutations in a subgroup derived from a mixture of BLCA, BRCA, UCEC, and LUAD tumors where all the samples were labeled as CIMP−. By contrast, within these same cancer types, amplification of a chromosomal region around *C17orf63* in tumors lacking amplification of *CCNE1* was observed in a subgroup that contained a higher proportion of CIMP+ than CIMP− labels. Other copy number events, such as amplification of the region containing *NKX2* and *FOXA1* or deletion of *HERC2* were observed in subgroups with a majority of CIMP+ samples and a large fraction of LUAD but also a few BRCA tumors. Deletion of a region containing *GALR1* was observed in a subset with a majority of CIMP+ tumors that came primarily from the COAD and HNSC types.

Our pan-cancer regression tree shows that *VHL* mutations correlate with significant reductions in average levels of CGI methylation in KIRC tumors (Additional file 1: Figure S2). Similarly, amplification of two chromosomal regions in chromosome 17, including *ERBB2* (a.k.a. *HER2*), co-occurs with an overall increase in CGI methylation in a subgroup consisting mostly of BRCA samples with some LUSC representation. Mutations observed in the context of decreased average methylation are *NSD1* in HNSC and *KDM6A* (sharing a mixed subgroup with a majority of BLCA tumors).

Individual tumor trees were also associated with recurrent functional events. For example, our classification tree for BLCA highlights alterations affecting *RB1* and *ARID1A* in CIMP+ tumors (Additional file 1: Figure S3), consistent with previous independent analyses [19,41]. In BRCA, we found a strong association between *CCND1* amplification and CIMP status (Additional file 1: Figures S3 and S4). Also, the presence of *MYC* amplifications delineated a subset of samples that consisted entirely of CIMP− tumors (Additional file 1: Figure S3). This is consistent with reports from TCGA identifying *MYC* amplification and high-expression in basal-like breast tumors, which tend to be hypomethylated [42]. In KIRC, the presence of either mutations or deletions affecting gene *SETD2* and methylation of the *GSTP1* promoter correlate with an important increase in the frequency of CIMP+ cases (Additional file 1: Figures S3 and S4). Also in KIRC, we found that deletion of a genomic region containing *CDKN2A* and *CDKN2B* on chromosome 9 is associated with increased levels of CGI methylation (Additional file 1: Figure S4). This kind of deletion has been linked to a more aggressive phenotype of clear cell carcinoma [43]. In LUSC, methylation of the *RBP1* promoter and amplification of a region containing *KDM5A* correlate with an increase in average CGI methylation (Additional file 1: Figure S4). In UCEC, our data show that methylation of the *MLH1* promoter results in a very high probability of CIMP+ status. For samples that do not exhibit this trait, the presence of *TP53* mutations is associated with the opposite outcome. Among the remaining samples, *PIK3R1* mutations are linked to increased CIMP+ rates (Additional file 1: Figure S3). Thus, the presence of tumor-specific mutations provides a potential link to predicting methylation status.

### Analysis of associations between methylator phenotypes and clinical features

**Figure 8:**
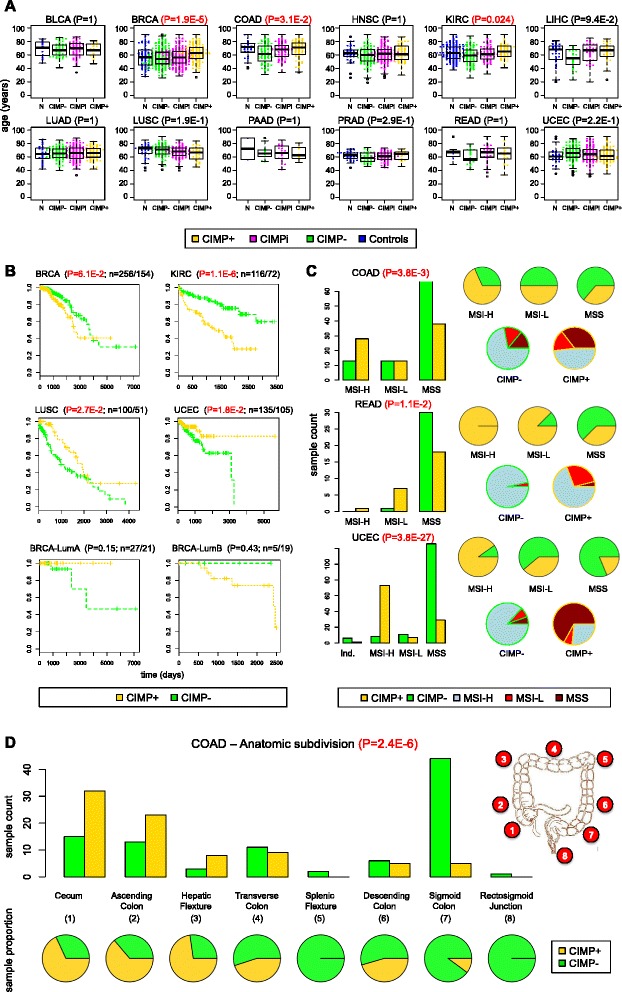
Associations between CIMP status and clinical annotations. **(A)** Age *vs*. CIMP status across 12 cancer types. **(B)** Overall survival curves for the four cancer types exhibiting significant differences based on CIMP status (BRCA, KIRC, LUSC, UCEC) and overall survival curves for luminal A and luminal B subtypes in BRCA based on CIMP status. **(C)** Microsatellite instability *vs*. CIMP status in COAD, READ, and UCEC. **(D)** CIMP status as a function of anatomic subdivision in COAD. *P*-values come from a Kruskal-Wallis test for difference in medians in panel **(A)**, a log-rank test for survival curve differences in panel **(B)**, and Fisher’s exact test in panels **(C)** and **(D)**. For each survival curve in **(B)**, the number of CIMP−/CIMP+ samples is provided next to the corresponding *P*-value.

We compared our sets of data-driven CIMP labels with clinical annotations provided by TCGA for individual samples. First, we note that the sets of controls used in our analysis covered a range of ages that is similar to the range of ages covered by the sets of tumor samples for most cancer types (Figure 8A). This fact rules out age effects as a confounding factor because our feature selection algorithm requires selected differentially methylated probe sites to remain consistently low across controls, leading to large overlaps in the ages associated with the three CIMP categories (Figure 8A). In fact, ANOVA results using the Kruskal-Wallis test fail to reject the null hypothesis of equal median ages for different CIMP subgroups in 9 out of 12 types (after applying Holm’s correction for multiple hypotheses). The only exceptions are BRCA, COAD, and KIRC. For these three types, the median age of CIMP+ patients is higher than the median age in CIMP− (consistent with an independent study of CIMP+ status in COAD [11]). We found no statistical association between CIMP status and gender in any cancer type, except KIRC (*P* = 0.025, Fisher’s exact test with Holm’s correction), where we observed a significantly higher frequency of CIMP+ labels in male samples (45%, 58 of 128) than in female samples (22%, 15 of 66).

Whereas mutational events are correlative, an association of survival and CIMP status would indicate relevant subclasses of tumors that could prompt more narrowly defined intervention strategies. We evaluated survival curves based on CIMP status for the 12 cancer types in our study and found significant differences in KIRC, LUSC, and UCEC (BRCA results were borderline significant at 0.06) (Figure 8B). CIMP+ samples exhibited better survival curves than CIMP− samples in UCEC and LUSC, whereas CIMP− samples exhibited better survival than CIMP+ in KIRC and BRCA. Of course, these results must be interpreted carefully because they result from a univariate analysis that does not explicitly take into account potentially confounding factors.

Consistent with prior knowledge, we also detected a significant level of statistical association between CIMP status and microsatellite instability (MSI) in the three cancer types for which this annotation was available (Figure 8C). These included COAD (*P* = 3.8 × 10^−3^; Fisher’s test with Holm’s correction), READ (*P* = 0.011), and UCEC (*P* = 3.8 × 10^−27^). In all cases, a vast majority of CIMP− samples were microsatellite stable while CIMP+ labels were more frequent among microsatellite instable samples. These results are consistent with independent reports of MSI in CIMP-high colorectal tumors [37] and also with the division between UCEC serous and endometrioid samples (largely CIMP− and CIMP+ in our analysis, respectively), where endometrioid tumors carry microsatellite instability and serous tumors do not [26]. Thus, the MSI characteristic appears to be associated with CIMP+ status and mutually exclusive with *TP53* mutations and copy number variation.

We observed statistically significant levels of association between our CIMP labels and the five categories commonly used to catalogue breast tumors [42] (Additional file 1: Figure S5A; *P* = 3 × 10^−8^, Fisher’s exact test). In effect, the basal and normal-like categories were enriched in CIMP− samples, the luminal B category consisted mostly of CIMP+ and the luminal A group contained a more balanced mixture of CIMP types. This conclusion is consistent with trends reported by TCGA, where many luminal B samples showed a hypermethylator phenotype while basal-like samples were hypomethylated and associated with very high rates of *TP53* mutations [42]. Still, treatment approaches for breast cancer often consider hormone responsiveness over subtype classification. In BRCA samples (Additional file 1: Figure S5A), CIMP+ appeared more frequently in ER+ samples than in ER− samples (*P* = 6.7 × 10^−5^, Fisher’s exact test, Bonferroni correction). Likewise, a large number of HER2+ tumors were CIMP+, suggesting applications for methylation inhibitors in combination therapy of CIMP+ tumors. In contrast, a majority of HER2− samples were CIMP− (*P* = 0.001, Fisher’s exact test, Bonferroni correction).

In COAD (Figure 8D), we observed a strong association between CIMP status and anatomic neoplasm subdivision (*P* = 2.4 × 10^−6^, Fisher’s exact test, Bonferroni correction). More precisely, there is a progressive decrease in the frequency of CIMP+ samples along the intestinal tract from cecum, through ascending, transversal, and descending colon and finishing at the rectosigmoid junction. This variation appears to be proportional to distance along the intestinal tract and is consistent with a previous study of colorectal cancer samples collected from three anatomic locations and assessed at eight CIMP-specific promoters using MethyLight technology [44], as well as independent reports of a gradual decrease in the frequency of *BRAF* mutations and microsatellite instability within this same region of the intestinal tract [45].

In HNSC (Additional file 1: Figure S5B), we also observed a significant level of association between CIMP labels and anatomic subdivision (*P* = 5.0 × 10^−3^, Fisher’s exact test, Bonferroni correction), with more CIMP+ than CIMP− samples at the oral cavity, buccal mucosa, and floor of the mouth. However, CIMP− labels outnumbered CIMP+ labels in the base of tongue, alveolar ridge, tonsil, and larynx. Also in HNSC, CIMP− samples exhibited significantly better survival curves for recurrence free status than CIMP+ samples (Additional file 1: Figure S5B).

In KIRC (Additional file 1: Figure S6A), samples labeled as CIMP+ tended to have higher grade (*P* = 2.5 × 10^−6^, Fisher’s exact test, Bonferroni correction) and higher pathological stage (*P* = 1.5 × 10^−12^, Fisher’s exact test, Bonferroni correction). They also exhibited higher T parameters (*P* = 8.4 × 10^−12^, Fisher’s exact test, Bonferroni correction) and M parameters (*P* = 5.6 × 10^−4^, Fisher’s exact test, Bonferroni correction) based on the TMN cancer staging notation system, which implies larger primary tumor sizes and higher distant metastatic spread, respectively. Together with the survival curves shown in Figure 8B, these indicators suggest a worse prognosis for CIMP+ patients than CIMP− patients in KIRC. This is consistent with our finding of recurrent *CDKN2A* and *CDKN2B* deletions in CIMP+ samples from KIRC patients mentioned earlier (Additional file 1: Figure S4), which were independently linked to a more clinically aggressive phenotype of kidney clear cell carcinoma [43].

In UCEC, we observed a strong association between CIMP status and histological subtype, wherein all the 68 samples of serous subtype had CIMP− labels and 103 of 161 endometrioid samples had CIMP+ labels, with 58 endometrioid samples being labeled as CIMP− (Additional file 1: Figure S6B, *P* = 9 × 10^−24^, Fisher’s exact test). These observations agree with our previous finding of a methylator phenotype occurring in endometrioid endometrial tumors but not serous endometrial tumors [25], as well as results reported by TCGA [26]. In fact, the association between CIMP status and histological subtype extends to tumor grade (an indicator of how quickly a tumor is likely to grow and spread based on microscopic appearance), where CIMP− samples exhibit higher grades than CIMP+ samples (Additional file 1: Figure S6B, *P* = 9.6 × 10^−4^, Fisher’s exact test, Bonferroni correction). In particular, all the high-grade samples were CIMP−, consistent with the fact that serous endometrial tumors tend to present higher grades than endometrioid endometrial tumors [46].

## Discussion

Overall, our results support the existence of both commonalities and tissue-specific differences in CGI hypermethylation patterns across tumors. The most important similarity found in our analysis is the existence of consistent levels of average CGI hypermethylation that correlate with CIMP status and are independent of cancer type (Figure 2B,C). A consequence of this is the identification of a pan-cancer set of 89 genomic loci that can accurately separate CIMP+ from CIMP− samples across 12 different cancer types. Our genome-wide analyses (Additional file 1: Figure S1) show that much of the focal, cancer-related CGI hypermethylation occurs at loci that exhibit consistently baseline levels of methylation in control samples. This finding is particularly obvious and relevant for gene promoters (Additional file 1: Figure S7A). These data support a model whereby CIMP arises through mechanisms of *de novo* methylation that are largely reproducible events in the genome, rather than random spontaneous events. Furthermore, our data show unequivocally that this happens at a large number of genomic regions in a coordinated manner (Figure 1). Importantly, CGI methylation within gene bodies reveals that targets of hypermethylation in CIMP+ tumors are also found outside of promoters (Additional file 1: Figures S7B,C).

Our results validate previous biological CIMP findings while unveiling potentially interesting new avenues of research. For example, significantly recurrent functional events in CIMP+ samples correspond to mutated genes or silencing of *MLH1* and *MGMT*, while recurrent events in CIMP− consist primarily of chromosomal amplifications and *TP53* mutations (Table 2). This suggests a possible, previously unreported pan-cancer correspondence between the mutually exclusive M class and C class (groups dominated by mutations and copy number changes) as defined in Ciriello *et al*. [27] and the CIMP+ and CIMP− categories, respectively. Similarly, our analysis of upstream regulators that are shared across different sets of differentially methylated probes points to important members of the PRC such as *EZH2* and *SUZ12*. This is consistent with previous reports of PRC targets being affected in CIMP tumors across cancers [47], but our results involve a much larger collection of cancer types. While mutations in gene *H3F3A*, which encodes histone variant H3.3, have been correlated with specific DNA methylation subgroups in pediatric glioblastoma [19], our analysis of upstream regulators implicates involvement in most of the cancer types that we evaluated, with the exception of LUAD and BLCA (Figure 3B). The same upstream analysis suggests potential relevance in CIMP of several members of the sonic hedgehog pathway, which is consistent with tissue-specific patterns of aberrant CGI methylation. Loss-of-function mutations in the demethylating enzyme *TET2* have been previously associated to CIMP in leukemia, and our results reveal recurrence of this mutation in CIMP+ for other types such as UCEC and READ (Figure 6A). Also, mutations of *ARID1A* have been linked to MSI and CIMP in gastrointestinal cancers, and our results indicate importance in UCEC and BLCA. *BRAF* mutations, which are perhaps one of the most commonly accepted indicator events for CIMP in colorectal cancers, also appear to be relevant in LUAD, but not the other tumor types.

Some of our results translate into biologically plausible hypotheses that could lead to refined treatment regimes. For example, amplification of genes *PIK3CA* and *CCNE1* occur significantly more frequently in CIMP− samples. Interestingly, both *PIK3CA* and *CCNE1* are directly or indirectly drug targetable [27], suggesting a possible combinational therapy aimed at CIMP− patients. Our pan-cancer regression tree revealed global CGI hypomethylation in samples with mutated *NSD1*, which came primarily from the HNSC data set (Additional file 1: Figure S2). The connection between *NSD1* and DNA hypomethylation is likely related to a loss of its histone H3K36 methyltransferase activity that is a documented event in Sotos syndrome [48-51]. Our results also linked mutations in *KDM6A*, a H3K27me3 demethylase, to decreased CpG island methylation (Additional file 1: Figure S2). Notably, H3K27me3 recruits the polycomb repressive complex to specific targets, including HOX genes whose regulation is critical during cell-differentiation [52]. Furthermore, H3K27me3 has been proposed to ‘pre-mark’ genes for *de novo* methylation in cancer by favoring the aberrant recruitment of DNA methyltransferases [53], which suggests that *KDM6A* mutations may play an important role for the establishment of CIMP. Along the same lines, amplification of histone demethylase *KDM5A*, which targets H3K4me3/me2 active marks [54,55], was shown in our LUSC regression tree as exhibiting significant correlation with variations in average levels of CGI methylation (Additional file 1: Figure S4).

The results from our analysis of clinical annotations reveal ways in which our sample stratification can be used to refine current molecular subtyping. For example, an early study of DNA methylation patterns in breast cancer by Fang *et al*. had reported good survival for CIMP+ tumors [47]. However, more recent analyses have linked luminal B tumors, which are generally characterized by high levels of CGI methylation, with poor survival [56,57]. Our results help to clarify this apparent contradiction by showing that poor survival could be associated with luminal B patients with CIMP+ status and that luminal B patients with CIMP− can have good survival outcomes (Figure 8B). Interestingly, the situation is reversed in luminal A tumors, where CIMP+ status is associated to good survival and CIMP− status is associated with poor survival (as originally reported by Fang *et al*.). The lack of statistical significance for the reported differences might be due to the small number of available samples (for example, only five samples were available in the ‘luminal B & CIMP−’ category), so the strong magnitude of effect that we observe should be re-evaluated in the context of a larger cohort. If confirmed, these results would suggest that the CIMP+/− and the luminal A/luminal B categorizations can be combined to build improved prognosis indicators and also that current therapies are better suited for treating patients in the ‘luminal B & CIMP−’ or ‘luminal A & CIMP+’ categories than they are for treating patients in the ‘luminal B & CIMP+’ or ‘luminal A & CIMP−’ subgroups. This is a circumstance that needs to be further investigated.

## Conclusions

Our tissue-specific selection of differentially methylated probes was important to identify concerted changes in average methylation levels that occur on top of the epigenetic background of tissue-specific repressive events. We have shown that those concerted changes are relevant for defining phenotypic and clinical differences among tumor samples. The cross-cancer analysis that we present also suggests that tissue-specific patterns may obscure detection of underlying pan-cancer epigenetic signals, which are often weak in comparison to competing signatures of cellular differentiation. Still, our results highlight the existence of several functional events that are relevant for CIMP across multiple cancer types and our set of 89 signature loci represents the first evidence for a pan-cancer methylation signature that can be used to classify multiple tumor types according to CIMP status.

We provide a robust, principled molecular stratification of solid tumors and cell lines based on CIMP signatures that can be reused in future studies to refine current molecular subtypes in a wide variety of cancers. By applying the same computational pipeline to samples from different tissues and cancer types, our work facilitates biologically meaningful cross-cancer comparisons. The many statistically significant associations between CIMP status and both genomic and clinical features that we report in our work show that our CIMP+ and CIMP− labels define biologically distinct subpopulations whose phenotypic differences transcend DNA methylation patterns. Beyond several findings that characterize CIMP status in a tissue-specific manner, our study highlights the existence of important commonalities underlying CIMP as a pan-cancer epigenomic phenomenon. Still, our results are mostly correlational in nature and the identification of a unifying mechanism for CIMP across cancer types remains elusive. In order to further characterize causal genomic alterations that drive CIMP while answering the question of whether CIMP itself is a driver or a passenger trait for tumorigenesis and cancer progression, future pan-cancer studies shall benefit from extended experimental frameworks that include large scale interventions based on refined tumor stratification.

## Methods

### Data

#### DNA methylation data from TCGA

We downloaded level 3 data for 15 different cancer types from the TCGA data portal (https://tcga-data.nci.nih.gov/tcga/). Data had been acquired using the Illumina HumanMethylation450K platform and had been pre-processed following TCGA standard protocols. Data were downloaded in October 2013. The number of tumor and control samples that we downloaded for each cancer type is shown in Table 1. For the colorectal (COREAD) validation experiment, where we compared our sample classification algorithm with the methylation clusters defined by TCGA [24], we used a separate data set from the Illumina HumanMethylation27K platform. Specifically, we combined all the 320 samples from the COAD and READ cancer types for which both Illumina HumanMethylation27K methylation data and methylation cluster labels (CIMPH, CIMPL, Cluster3, Cluster4) were available.

##### Data pre-processing

The data that we used had gone through all the pre-processing associated with level 3 data from TCGA. We discarded all the probes that interrogated locations in chromosomes X and Y, as well as all probes that were masked as NA (‘Not Available’) for more than 90% of the samples. In the case of the KIRP dataset, we excluded nine tumor samples that behaved as outliers based on PCA plots computed over variably methylated probes (these tumor samples clustered together with each other, away from the rest of tumors and closer to the set of controls; the actual sample IDs were TCGA-A4-7915-01, TCGA-F9-A4JJ-01, TCGA-G7-6793-01, TCGA-GL-7966-01, TCGA-P4-A5E8-01, TCGA-P4-A5EA-01, TCGA-BQ-5879-01, TCGA-BQ-5893-01, TCGA-BQ-5894-01). We normalized the data individually for every sample in every cancer type using the BMIQ method [58], which corrects for technical differences between type I probes and type II probes in the Illumina HumanMethylation platform. This method was chosen based on positive reviews from a recent study that compared several normalization methods [59]. A more detailed analysis of the technical biases associated with different Illumina probe types and their effect upon our probe selection criterion is provided in Additional file 2. We also provide an exploratory analysis of the impact of batch effects upon our sample stratification in Additional file 2.

#### DNA methylation data from ENCODE

We used DNA methylation data for 51 human cell lines from ENCODE (Additional file 5: Table S3). The data were downloaded from the HAIB Methyl450K track of the UCSC Human Genome Browser (https://genome.ucsc.edu/) and had originally acquired using the Illumina HumanMethylation450K Bead Array platform, as was the case for the solid epithelial tumors data from TCGA. Data was pre-processed following the same guidelines that we had described for solid epithelial tumors.

#### RNA-Seq data for TCGA samples

Gene expression RNA-Seq data was downloaded from the TCGA data portal between January and June 2014 (Additional file 5: Table S5). We used RNA-Seq V2 data processed at level 3. We used the files ending with ‘rsem.genes.normalized_results’ - these files contain gene expression values for 20,531 genes. Gene expression in these files is normalized so that the third quartile of genes with positive expression is set to 1000, for each sample. We removed 29 genes that did not have a gene symbol. The COAD, READ, and UCEC data sets contained data from both the GA and HiSeq sequencing platforms. We merged these data as follows: if a given sample was present on both platforms, we kept only the HiSeq version. If only the GA or HiSeq version was present, then it was kept. Additional file 5: Table S5 contains the resulting number of samples in each cancer type.

#### Selected functional event data for TCGA samples

We downloaded data for 479 selected functional events across 3,299 TCGA samples that were made publicly available by the cBio group at Memorial Sloan Kettering Cancer Center (http://cbio.mskcc.org/cancergenomics/pancan_tcga/), as described in Ciriello *et al.* [27]. We used the genomic alterations matrix file containing filtered calls only with date stamp of 5/31/2013.

#### Clinical data for TCGA samples

All the clinical data that we used in our analysis were downloaded from the UCSC Cancer Genomics Browser (https://genome-cancer.ucsc.edu/) [60]. All clinical data files had time stamp of 12/18/2013. The actual set of available annotations varied across cancer types. Also, within each cancer type, the set of available annotations varied across samples.

### Statistical methods

All our computations were done using the R statistical package (with the only exception of the *P*-values shown in Figure 3, which were computed directly using the IPA software). We used CpG island annotations from UCSC for hg19 and gene annotations provided by Illumina for their HumanMethylation450K platform.

#### Statistical significance and biological relevance for probe selection thresholds

Our approach to feature selection requires the use of two parameters that represent the maximum threshold for average methylation across controls (α_C_) and the minimum threshold for average methylation across tumors (α_T_). A probe will be selected for inclusion into the differentially methylated set if and only if its average level of methylation computed over all the control samples is below α_C_ and its average level of methylation computed over all the tumor samples is above α_T_. A choice of α_C_ = 0.05 and α_T_ = 0.25 seemed biologically reasonable to us in order to capture probes that exhibit consistently low levels of methylation in controls while presenting at least some sufficiently high level of methylation signal in tumors. We show statistical significance and biological relevance for this choice of thresholds:

- In order to evaluate statistical significance, we ran a random permutation experiment to estimate the number of false positive detections associated with this choice of parameters. More precisely, we considered each individual cancer type separately and we proceeded as follows: (1) we randomly shuffled the ‘control’ and ‘tumor’ labels for all the samples in the data set, (2) we applied our sample selection algorithm with parameters (*α*_C_ = 0.05, *α*_T_ = 0.25) to the randomly shuffled data, and (3) we counted the number of selected features. These counts represent the number of features selected under the null hypothesis of randomly labeled samples (that is, when tumor *vs*. control label assignment is independent of sample identifier), and therefore, they provide an estimate of the false positive rate associated to our feature selection procedure. The number of selected features averaged over 100 random permutations was below 0.1 for all types except READ (5.50) and STAD (502.32). The worst results were obtained for the cancer types with the lowest number of control samples, since this increases the probability of regions with low methylation being randomly aligned across those samples. When looking at our actual data, the number of selected features for READ was 1,255, which leads to an acceptably low false detection rate. In the case of STAD, however, the rate between expected false detections and actual detections was around 50% (502.32/1,110). Since only two controls were available for this cancer type at the time of our analysis, we found that any choice of threshold that guarantees a sufficiently low rate of false detections results in no differentially methylated probes being selected at all, so we decided to exclude STAD from most parts of our analysis.
- We evaluated biological relevance, in the sense of sufficiently large magnitude of effect. For this, we computed the average difference in mean per-probe methylation for samples in the CIMP+ *vs.* CIMP− category (where labels had been learned using *α*_C_ = 0.05 and *α*_T_ = 0.25) for each individual cancer type (Additional file 1: Figure S9). For every cancer type, we observe differences in beta values of at least 0.1 and 0.3 when the variably methylated set and the differentially methylated set, respectively, are used to estimate average per-probe methylation in the CIMP+ and CIMP− subsets of samples. These mean differences are large enough to be considered biologically relevant (the TCGA marker paper on ovarian cancer [61], for example, proposes to use mean per-probe differences of 0.1 and 0.3 between tumors and controls as a relaxed and stringent threshold, respectively, in order to establish gene hypermethylation, while others such as Ciriello *et al*. rely on a single hard threshold of 0.1 [27]).

#### Comparison to previously published hierarchical clustering results from TCGA

For validation purposes and to address whether our classifications correspond convincingly to known examples of CIMP phenotypes from the literature, we compared our method with previously published results from TCGA that were also based on hierarchical clustering of DNA methylation levels. We applied our sample classification algorithm to an independent set of 320 colorectal samples (233 tumors and 87 controls) that had been previously analyzed by the TCGA Network [24] (see the ‘Methods’ section). These samples were used only for validation purposes and consisted of a mixture of 240 COAD samples (165 tumors and 75 controls) and 80 READ samples (68 tumors and 12 controls). Only a small subset of these samples overlapped our pan-cancer analysis (that is, 4 tumors and 38 controls were present in COAD and 1 tumor and 7 controls were present in READ from the pan-cancer data sets). Nevertheless, the data for these 320 TGCA samples derived from the HumanMethylation27K platform in contrast to the HumanMethylation450K data depicted in Table 1, enforcing that no measurements were reused in the two separate analyses. TCGA classified these samples into four different clusters (CIMPH, CIMPL, Cluster3, Cluster4) based on their overall levels of DNA methylation. Clusters CIMPH and CIMPL were described as having higher rates of methylation than the other two clusters. Our algorithm identified 86 samples as CIMP+, which all belonged to the CIMPH or CIMPL clusters (Additional file 1: Figure S10). Additionally, we identified 59 samples as CIMP−, which all belonged to the Cluster3 or Cluster4 categories. The remaining 88 tumors classified as CIMPi. The contingency table comparing the results from the two classification algorithms yielded a highly significant level of association based on Fisher’s exact test (*P* = 2.15 × 10^−67^).

#### Selection of pan-cancer differentially expressed genes in CIMP

We evaluated individually each of the 3,892 CIMP + ^Hyper^ and 54 CIMP + _Hypo_ regions that were associated to at least one known gene to search for significant correlations between DNA methylation and gene expression. In fact, since some of these regions were associated to more than one gene, we evaluated a total of 4,840 gene-region pairs. For each pair consisting of a gene and a CIMP + ^Hyper^/_Hypo_ region, we computed the Spearman correlation between the average level of methylation measured by each individual probe in the region and the RNA-Seq level of expression measured for the gene. We then selected the probe with the highest absolute coefficient of correlation as the cluster representative. We did this separately for each of the 12 cancer types in our analysis. Figure 5B shows the set of 121 genomic regions and 93 genes that exhibited significant levels of correlation (FDR < 0.10) for all 12 cancer types.

#### Analysis of selected functional events from Ciriello *et al*.

Our analysis of differential frequencies in CIMP+ *vs*. CIMP− samples was done by counting the number of samples in each of the two CIMP categories that presented each SFE for each individual type of cancer. Those counts were normalized by the total number of CIMP+ and CIMP− samples in each cancer type in order to turn them into frequencies of occurrence. For each SFE and each cancer type, we subtracted the frequency of occurrence in CIMP− samples from the frequency of occurrence in CIMP+ samples and we applied hierarchical clustering to draw the heat map shown in Figure 6A. We then pooled together all samples across different cancer types, and we computed global pan-cancer counts of occurrence for each selected functional event within the CIMP+ and the CIMP− subpopulations. We performed Fisher’s exact test to evaluate associations between CIMP labels and sample counts for each individual SFE. We ranked the samples in terms of increasing *P*-values and we showed the top 20 scorers in Table 2 (including FDR values to correct for multiple hypothesis testing). For the comparison of the average number of mutation, amplification and deletion events per sample shown in Figure 6B, we provide a bar plot showing mean number of events of each category for each individual cancer type. Error bars show 95% confidence intervals centered at the estimated means. The *P*-values shown in the figure correspond to a one-sided *t*-test. Finally, our analysis using binary decision trees was done with the R package ‘partykit’ [62], which provides tools for working with tree models for classification and regression. In the classification case, we restricted the analysis to samples that had been previously labeled as CIMP+ or CIMP− and we used the CIMP status as class label. In the regression case, we worked with all the samples for which both methylation and SFEs data were available, and in the case of the pan-cancer tree (Additional file 1: Figure S2), we used the average level of methylation computed across all the probes in the pan-cancer union of variably methylated probe sets (for all the nine cancer types under consideration) as the response or dependent variable. Regression trees for individual cancer types (Additional file 1: Figure S4) were learned using the average level of methylation computed across all the probes in the cancer-specific variably methylated probe set as the response variable.

#### Analysis of clinical annotations

We divided our analysis of clinical annotations into two separate parts. First, we evaluated statistical associations between CIMP status and a number of clinical annotations that we considered inherently relevant to our study. This included age, gender, microsatellite instability, and overall survival. We evaluated associations between CIMP status and patient age at the time of diagnostic using the Kruskal-Wallis test for analysis of variance. We tested for statistical associations between CIMP labels and categorical clinical annotations using Fisher’s exact test. In all these cases, we used Holm’s correction for multiple hypotheses restricted to the number of cancer types tested for each individual annotation. We compared survival curves for CIMP+ *vs*. CIMP− tumors using the log-rank test (the *P*-values that we report for survival curve comparisons were not corrected for multiple hypotheses). For the second part of our study of clinical annotations, we did an exploratory analysis where we evaluated a set of 300 categorical clinical annotations, most of which were available only for a small subset of the 12 cancer types. In particular, we ran a total of 653 individual tests involving specific pairs of cancer type and annotation. Due to the exploratory nature of this part of our analysis, we applied the more conservative Bonferroni correction with a factor of 653 to correct for multiple hypothesis testing.

## Additional files

Additional file 1:
**Supplemental figures S1 to S12.**
Additional file 2:
**Supplemental methods.**
Additional file 3:
**Selected sets of differentially methylated probes for TCGA samples.**
Additional file 4
**CIMP labels for TCGA samples.**
Additional file 5:
**Supplemental tables S1 to S7.**

## Abbreviations

BLCA: bladder urothelial carcinoma
BRCA: breast invasive carcinoma
CGI: CpG island
CIMP−: CIMP negative
CIMP: CpG island methylator phenotype
CIMP+: CIMP positive
CIMPi: CIMP intermediate
COAD: colon adenocarcinoma
EMT: epithelial-mesenchymal transition
ENCODE: Encyclopedia of DNA Elements
HNSC: head and neck squamous cell carcinoma
IPA: Ingenuity Pathway Analysis
KIRC: kidney renal clear cell carcinoma
KIRP: kidney renal papillary cell carcinoma
LIHC: liver hepatocellular carcinoma
LOTO: leave-one-type-out
LUAD: lung adenocarcinoma
LUSC: lung squamous cell carcinoma
MSI: microsatellite instability
PAAD: pancreatic adenocarcinoma
PCA: principal component analysis
PRAD: prostate adenocarcinoma
PRC: polycomb repressor complex
READ: rectum adenocarcinoma
SD: standard deviation
SFE: selected functional event
STAD: stomach adenocarcinoma
TCGA: The Cancer Genome Atlas
THCA: thyroid carcinoma
UCEC: uterine corpus endometrioid carcinoma
