# Supplementary material for: Pan-cancer stratification of solid human epithelial tumors and cancer cell lines reveals commonalities and tissue-specific features of the CpG island methylator phenotype
Source: Epigenetics Chromatin. 2015 Apr 17;8:14. doi: 10.1186/s13072-015-0007-7 (PMC4424513; doi:10.1186/s13072-015-0007-7)

Pan-cancer stratification of solid human epithelial tumors and cancer cell lines reveals commonalities and tissue-specific features of the CpG island methylator phenotype

Francisco Sánchez-Vega, Valer Gotea, Gennady Margolin, Laura Elnitski[§]

Translational and Functional Genomics Branch, National Human Genome Research Institute, National Institutes of Health, Bethesda, Maryland, United States of America.

[§]Corresponding author

Email addresses:

FSV: sanchezf@cbio.mskcc.org

VG: vgotea@nih.gov

GM: Gennady.Margolin@nih.gov

LE: elnitski@mail.nih.gov

Supplemental Figures

Figure S1 - Average methylation and methylation variability at variably methylated sites in TCGA tumors *vs.* controls.

Figure S2 - Pan-cancer partitioning of TCGA tumors using a binary regression tree..

Figure S3 - Cancer type specific selection of relevant CIMP features and sample classification using binary decision trees.

Figure S4 - Cancer type specific selection of relevant CIMP features using binary regression trees.

Figure S5 - Additional associations between CIMP status and clinical annotations.

Figure S6 - Associations between CIMP status and clinical annotations in KIRC.

Figure S7 - Average methylation of varCGI probes at promoters and gene bodies for CIMP+ *vs.* CIMP- samples.

Figure S8 - Distribution of average sample methylation based on different subsets of probes.

Figure S9 - Differences in average per-probe DNA methylation for CIMP+ vs. CIMP- samples.

Figure S10 - Validation of CIMP+ and CIMP- labels by comparison to published results from TCGA.

Figure S11 - BMIQ normalization to correct for Illumina probe type biases.

Figure S12 - Exploratory analysis of batch effects upon CIMP status classification.

Figure S1 - Average methylation and methylation variability at variably methylated sites in TCGA tumors *vs.* controls. Probe density as a function of mean methylation and standard deviation in tumors vs. controls across cancer types. Densities were computed after discarding probes in chromosomes X and Y, as well as probes with low variance (we required SD<0.1). **(A)** Probe density as a function of mean methylation in tumors vs. controls across cancer types, for variably methylated probes located within CGIs. **(B)** Probe density as a function of standard deviation in tumors vs. controls across cancer types, for variably methylated probes located within CGIs. **(C)** Probe density as a function of mean methylation in tumors vs. controls across cancer types, for probes located in CGI shores and shelves. In all panels, color scale was normalized for each cancer type and ranges from dark blue (lowest density of probes) to dark red (highest density). The dotted white diagonal line represents equal values of average probe methylation in tumors and controls. Panels (A) and (B) show that, for most cancer types under consideration, there exists a set of variably methylated sites with very low levels of methylation in controls and widely varying levels of methylation across tumors. By contrast, panel (C) shows that probes located in CGI shores and shelves (i.e., in 2 kb flanking regions of CGIs and CGI shores, respectively) tend to exhibit high levels of methylation in controls and slightly decreased levels of methylation in tumors. More details about the way in which probe densities were computed and drawn are provided in Supplemental Methods 1.

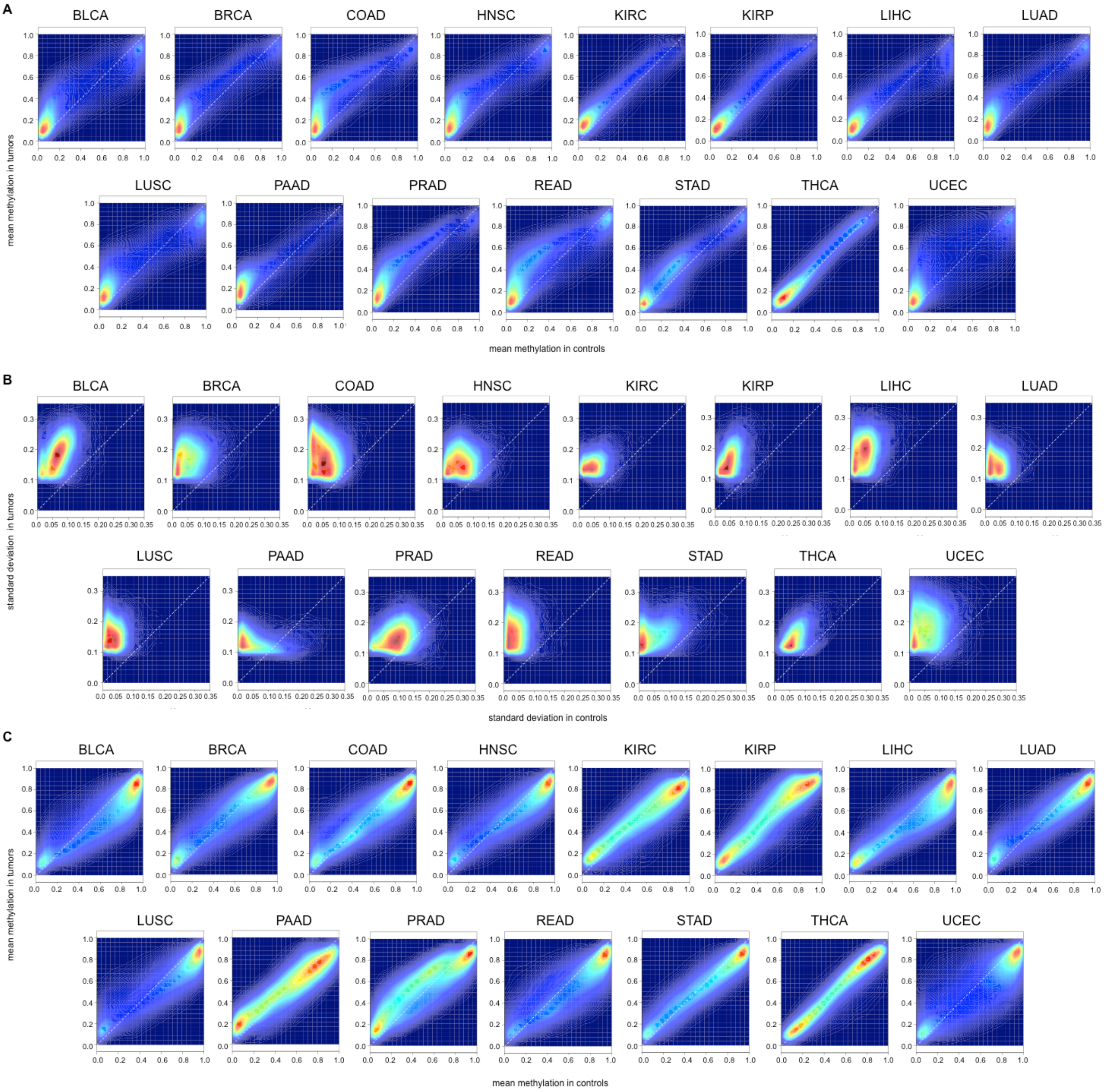

Figure S2 - Pan-cancer partitioning of TCGA tumors using a binary regression tree. Pan-cancer regression tree using average levels of CGI methylation computed over the union of variably methylated probe sets as the target response variable. Red and green branches illustrate absence or presence of the corresponding SFE, respectively. Terminal nodes show number of samples and associated boxplot of average methylation values, as well as proportions of different cancer types represented in each subset.

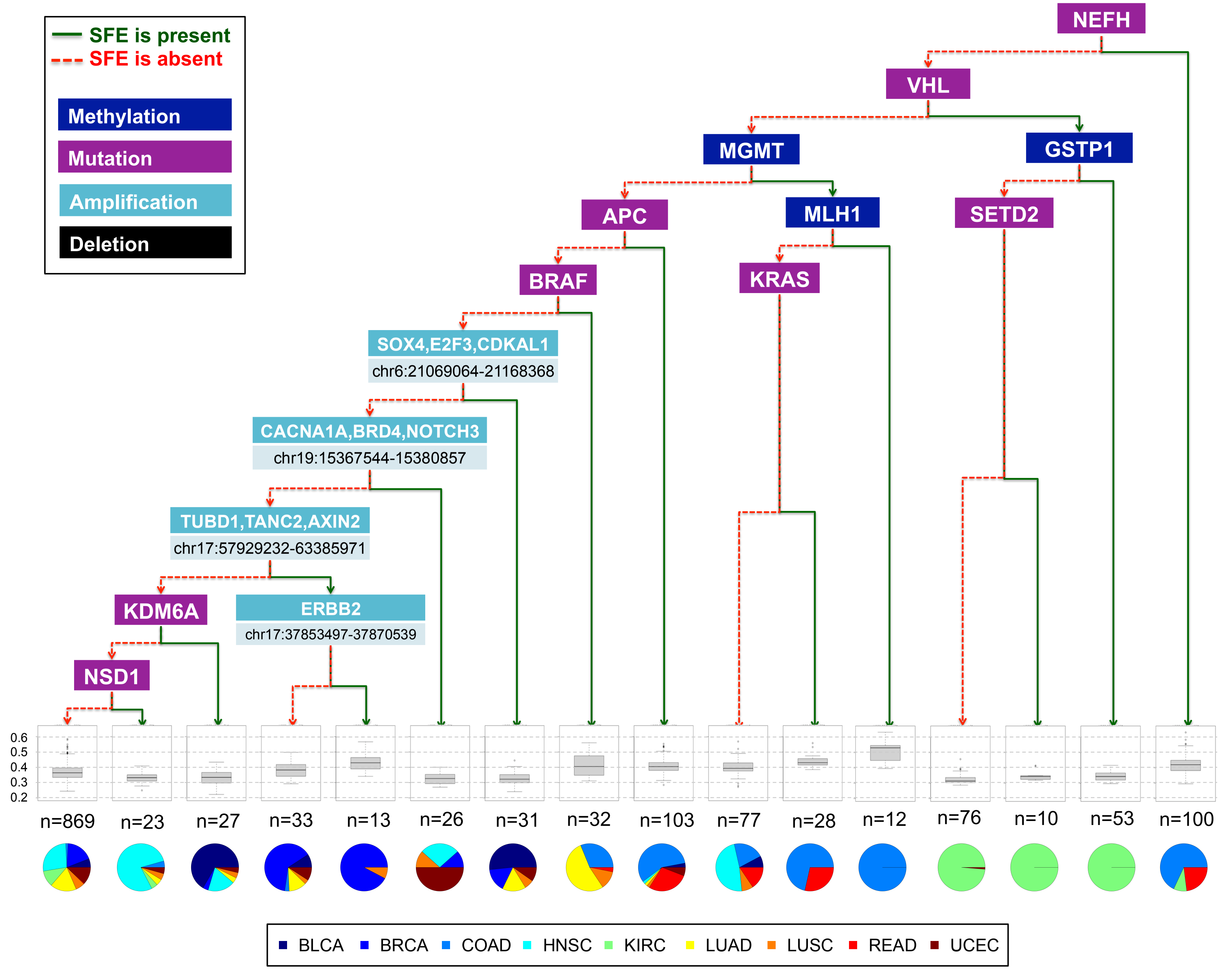

Figure S3 - Cancer type specific selection of relevant CIMP features and sample classification using binary decision trees. Binary trees for classification of tumor samples into the CIMP+ and the CIMP- category in individual cancer types. Terminal nodes show number of samples and associated proportion of CIMP+ *vs*. CIMP- labels. Red and green branches illustrate absence or presence of the corresponding SFE, respectively.

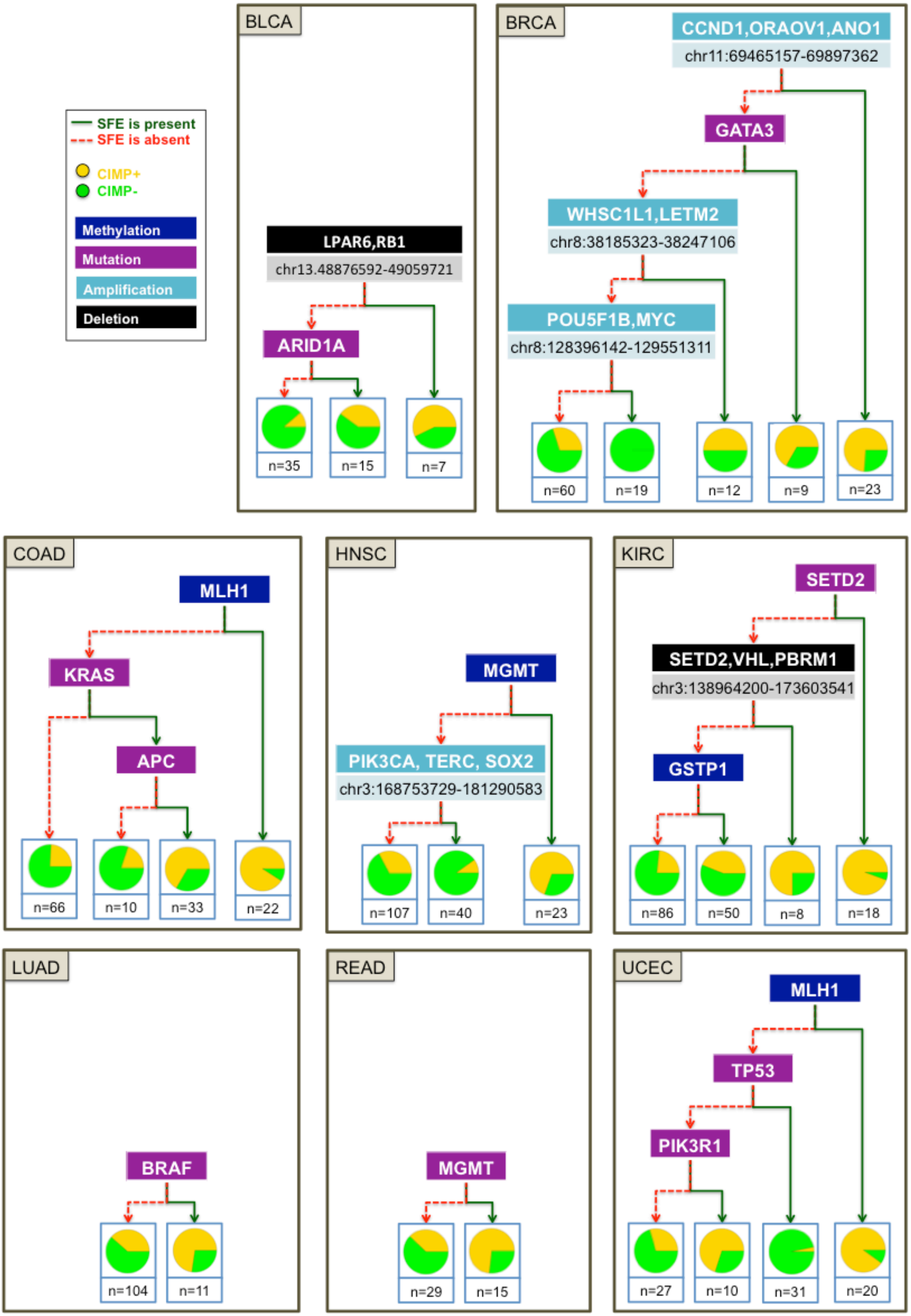

Figure S4 - Cancer type specific selection of relevant CIMP features using binary regression trees. Regression trees using average levels of CGI methylation computed over variably methylated probes as the target response variable in individual cancer types. Terminal nodes show number of samples and associated boxplot of average methylation values. Red and green branches illustrate absence or presence of the corresponding SFE, respectively.

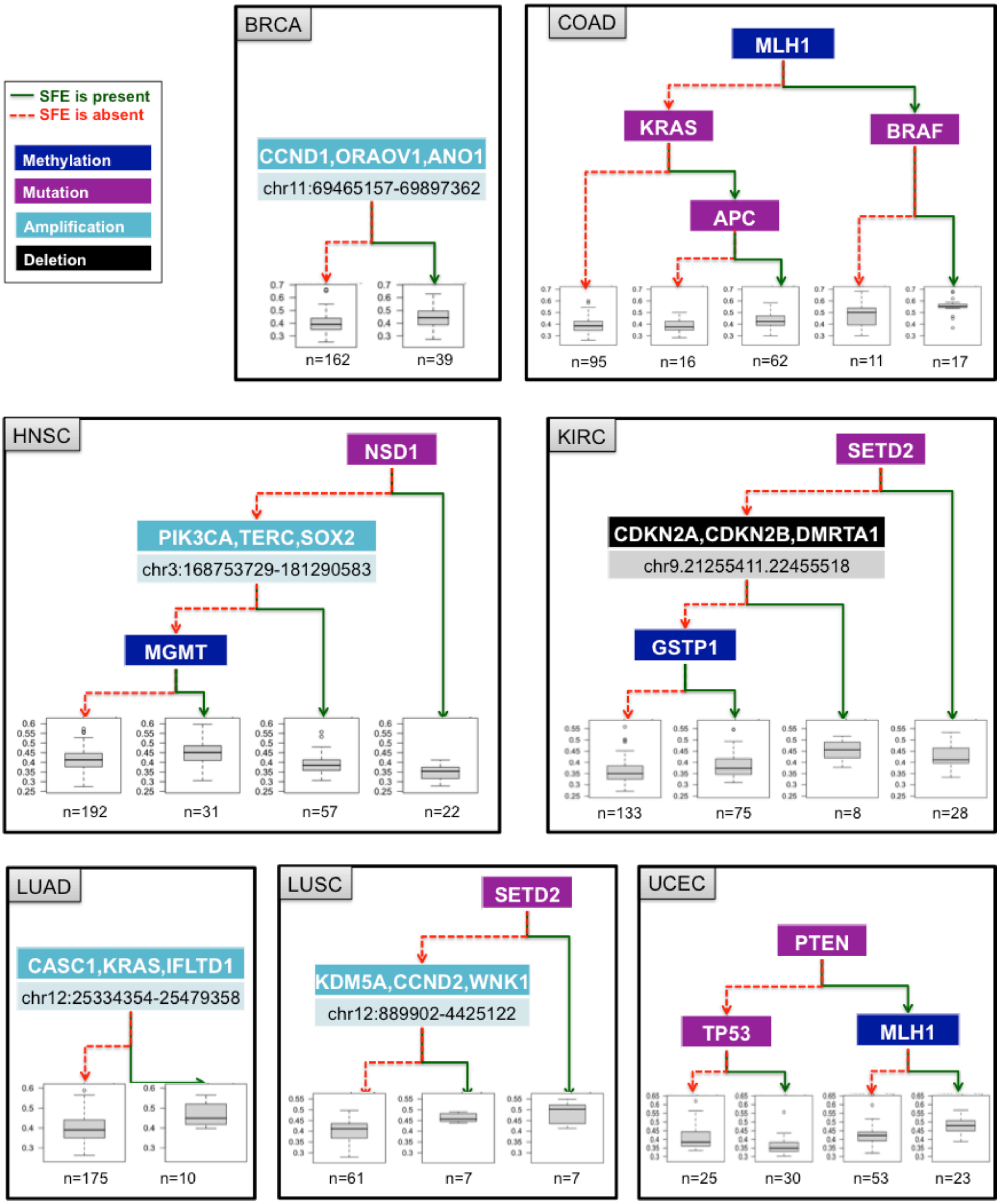

Figure S5 - Associations between CIMP status and clinical annotations in BRCA and HNSC. (A) PAM50 molecular subgroups, ER status and HER status *vs*. CIMP status in BRCA. **(B)** Anatomic subdivision and recurrence free survival curves as a function of CIMP status in HNSC. All p-values correspond to Fisher's exact test for statistical associations and were corrected for multiple hypotheses using Bonferroni correction (except the p-value for the survival curve, which comes from a log-rank test and is shown uncorrected).

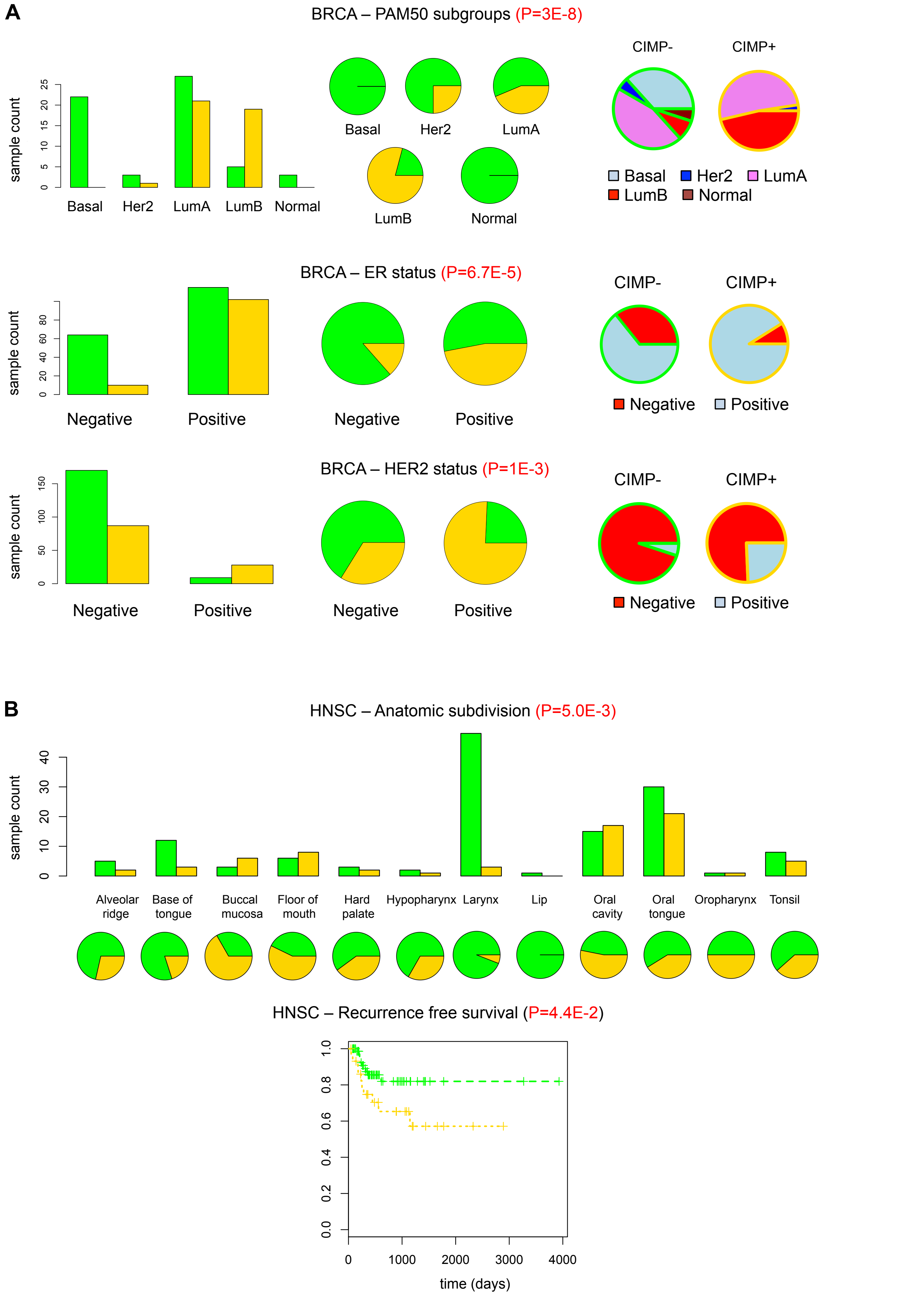

Figure S6 - Associations between CIMP status and clinical annotations in KIRC and UCEC. (A) Histologic grade, pathologic stage, pathologic M, and pathologic T *vs.* CIMP status in KIRC. (B) Histologic type and histologic grade *vs.* CIMP status in UCEC. All p-values correspond to a Fisher's exact test for statistical associations and were corrected for multiple hypotheses using Bonferroni correction.

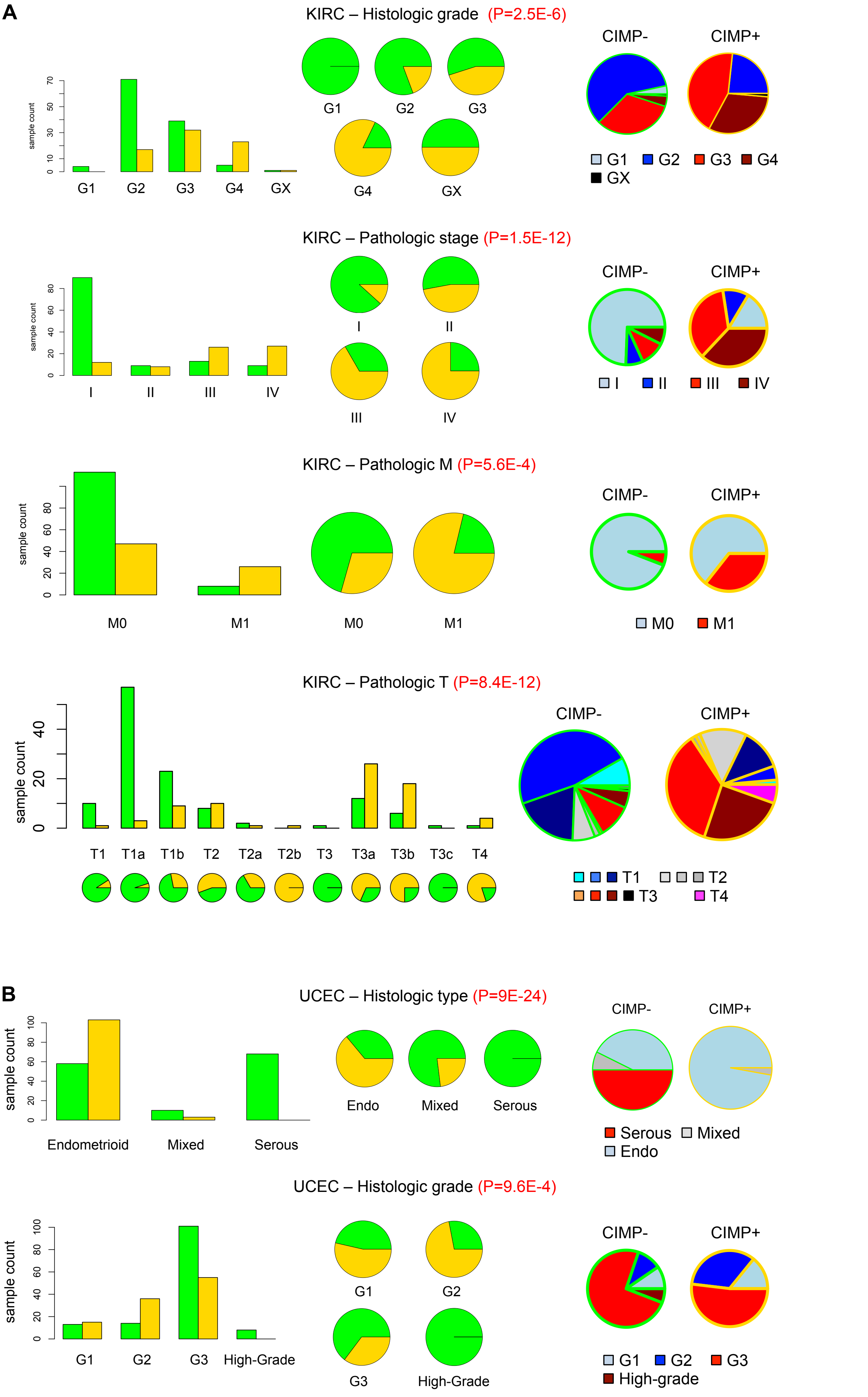

Figure S7 - Average methylation of variably methylated probes at promoters and gene bodies for CIMP+ *vs* CIMP- samples. (A) Density of variably methylated probes at gene promoters as a function of mean methylation in tumors *vs*. controls **(B)** Density of variably methylated probes at gene bodies (i.e., after the last nucleotide of the 1st exon and the last nucleotide of the corresponding 3'UTR) as a function of mean methylation in tumors *vs*. controls **(C)** Average per-sample methylation computed over the set of probes in promoters (horizontal axis, probes shown in panel A) *vs*. the average sample methylation computed over the set of probes in gene bodies (vertical axis, probes shown in panel B). Probes that were associated to both gene promoters and gene bodies were excluded from the plots, so that the two probe sets are mutually exclusive. Color scale was normalized for each cancer type and ranges from dark blue (lowest density of probes) to dark red (highest density). The dotted white diagonal line represents equal values of average probe methylation in tumors and controls.

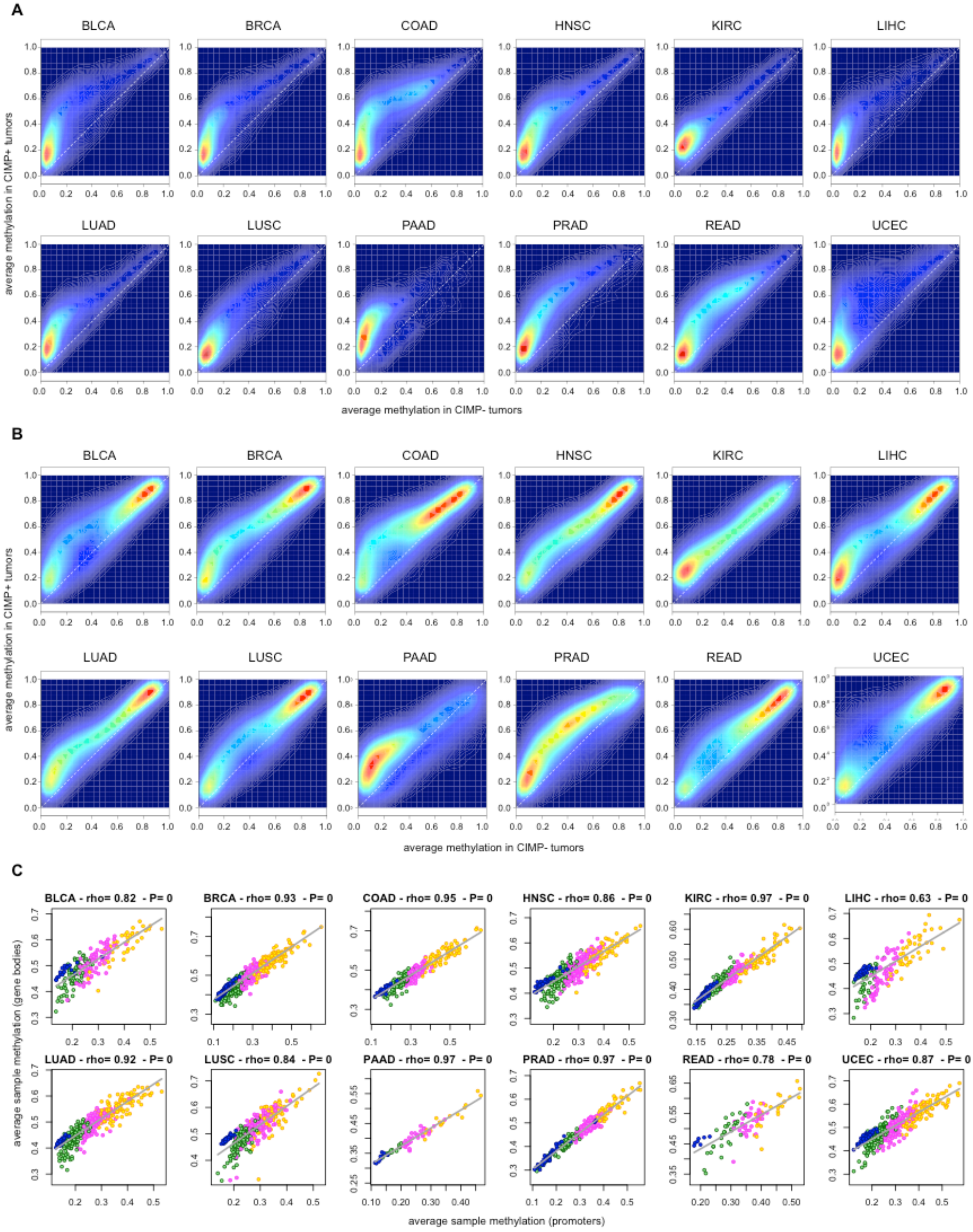

Figure S8 - Distribution of average sample methylation based on different subsets of probes. (A) Histogram of average sample methylation values computed over the entire set of CGI probes in the array for each cancer type. **(B)** Histogram of average sample methylation values computed over the entire set of variably methylated probes for each cancer type. **(C)** Histogram of average sample methylation values computed over the entire set of differentially methylated probes for each cancer type. In all panels, probes located in chromosomes X and Y were excluded from the analysis.

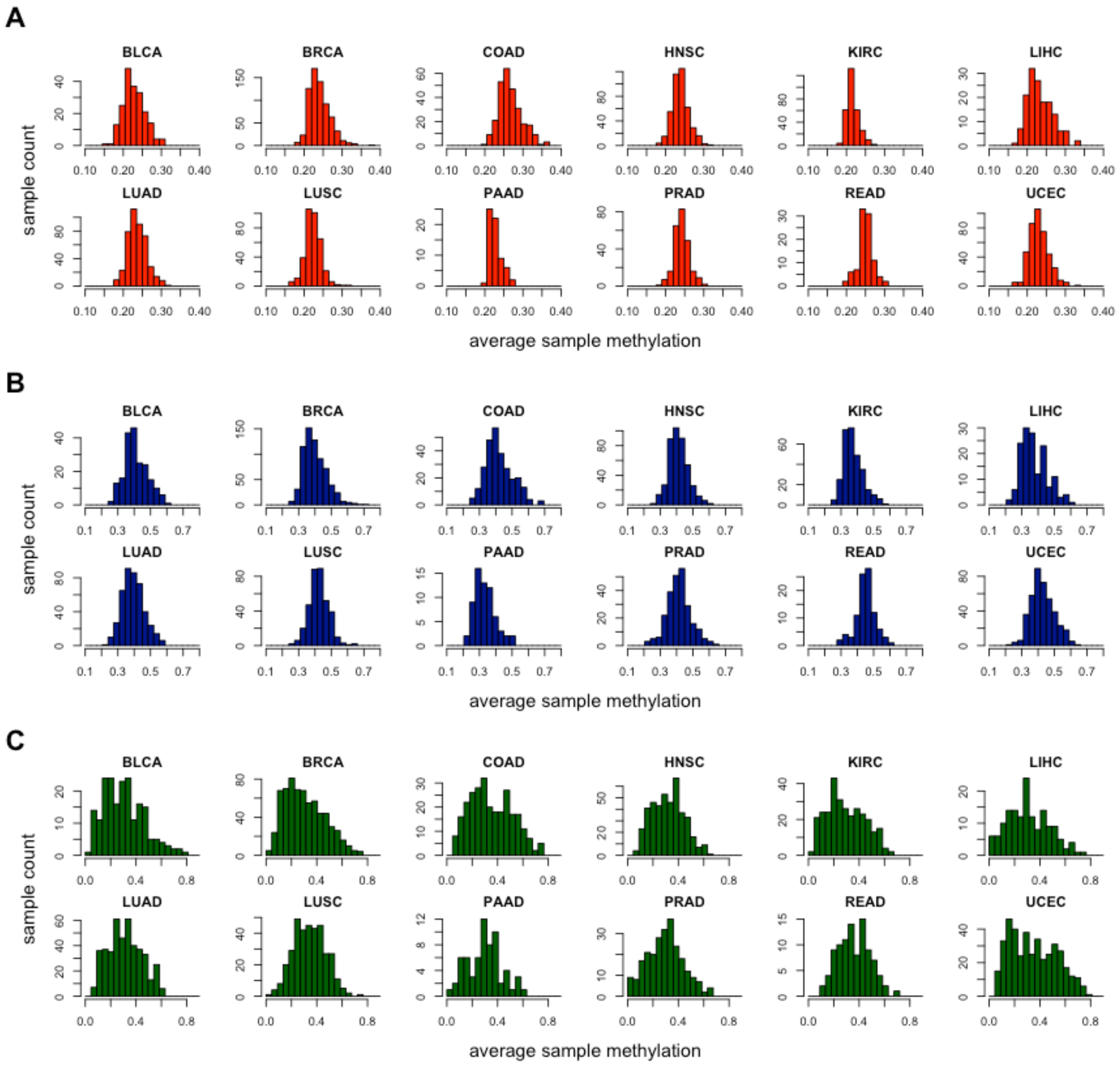

Figure S9 - Differences in average per-probe DNA methylation for CIMP+ vs. CIMP- samples. Vertical bars show average per-probe differences in DNA methylation in CIMP+ tumors minus CIMP- tumors. These mean difference levels were averaged over probes and over samples. Two sets of results are provided, computed over variably methylated and over differentially methylated probes, respectively. Error bars show 95% confidence intervals centered at the estimated means.

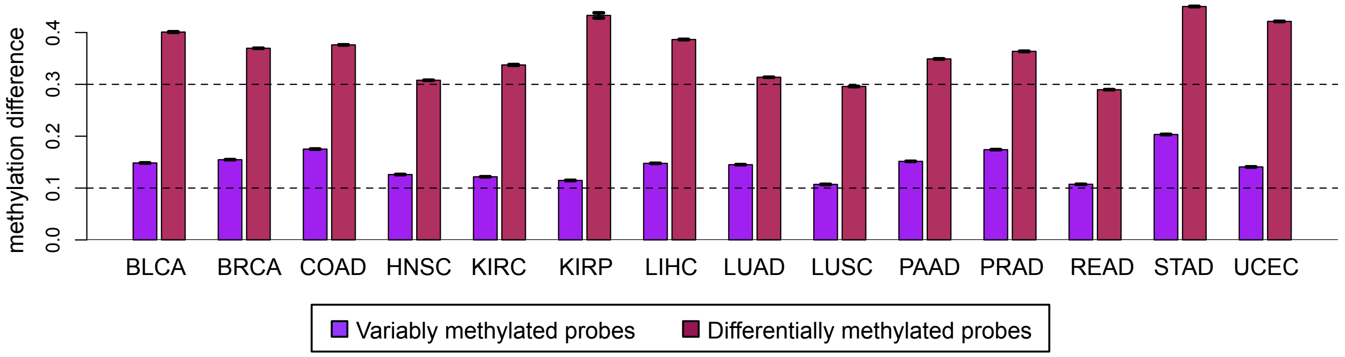

Figure S10 - Validation of CIMP+ and CIMP- labels by comparison to published results from TCGA. (A) Comparison of results from our sample classification algorithm and labels from the TCGA Network flagship paper on colorectal cancer. Rows and columns represent samples and differentially methylated probes, respectively. Rows were ranked from top to bottom in decreasing order of average methylation computed over differentially methylated probes. White dashed horizontal lines were used to highlight different CIMP subgroups based on the results from our classification algorithm. **(B)** Association between CIMP status and histologic type in UCEC tumors. Left panel shows total number of CIMP+ and CIMP- samples *vs*. histologic subtype. Middle and right panel show pie charts that illustrate proportion of CIMP labels for each histologic type and proportions of histologic types of each CIMP label, respectively. **(C)** Association between CIMP status and PAM50 subgroups in BRCA tumors.

A

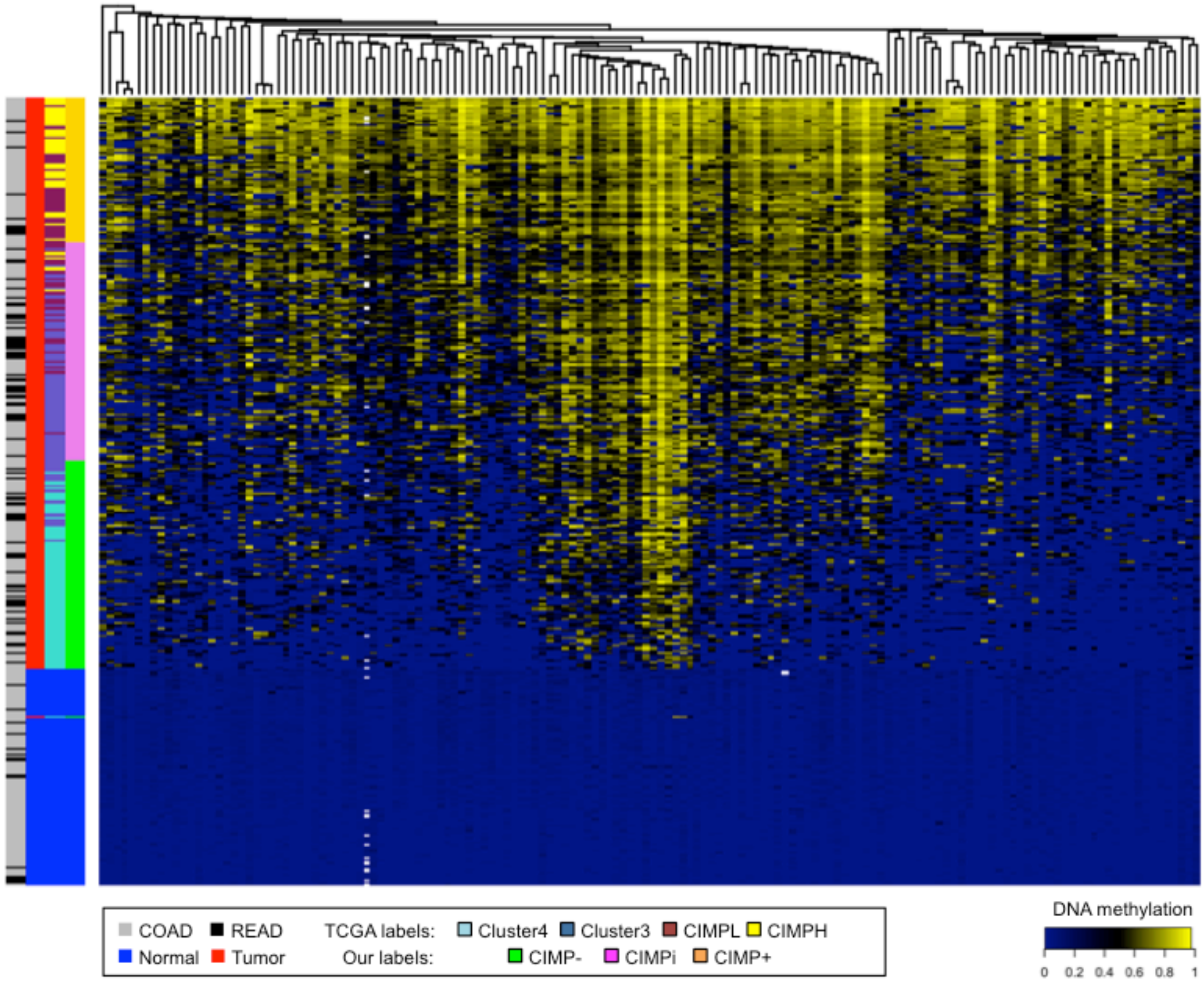

Figure S11 - BMIQ normalization to correct for Illumina probe type biases. (A) Number of Type I and Type II probes selected as differentially methylated probes before and after BMIQ correction (Type I probes are not corrected by this algorithm, so only one value is reported). **(B)** Average methylation computed over the set of Type I differentially methylated probes *vs*. average methylation computed over the set of Type II probes.

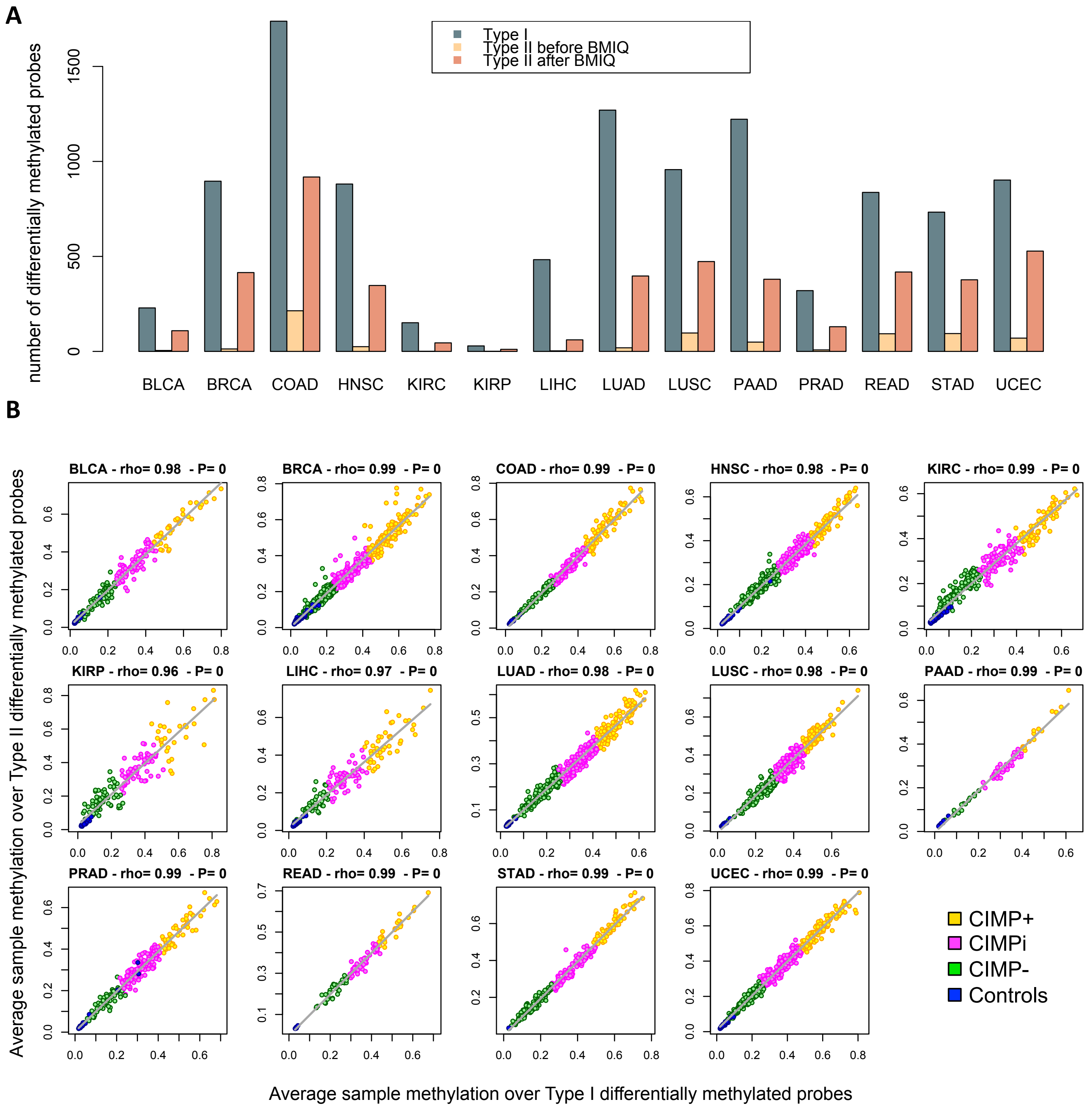

Figure S12 - Exploratory analysis of batch effects upon CIMP status classification For each cancer type, a stacked bar plot shows the number of samples labeled as CIMP+ and CIMP- (as well as controls) that belong to each different batch. Labels on the horizontal axis represent individual batch ids, as provided by TCGA.

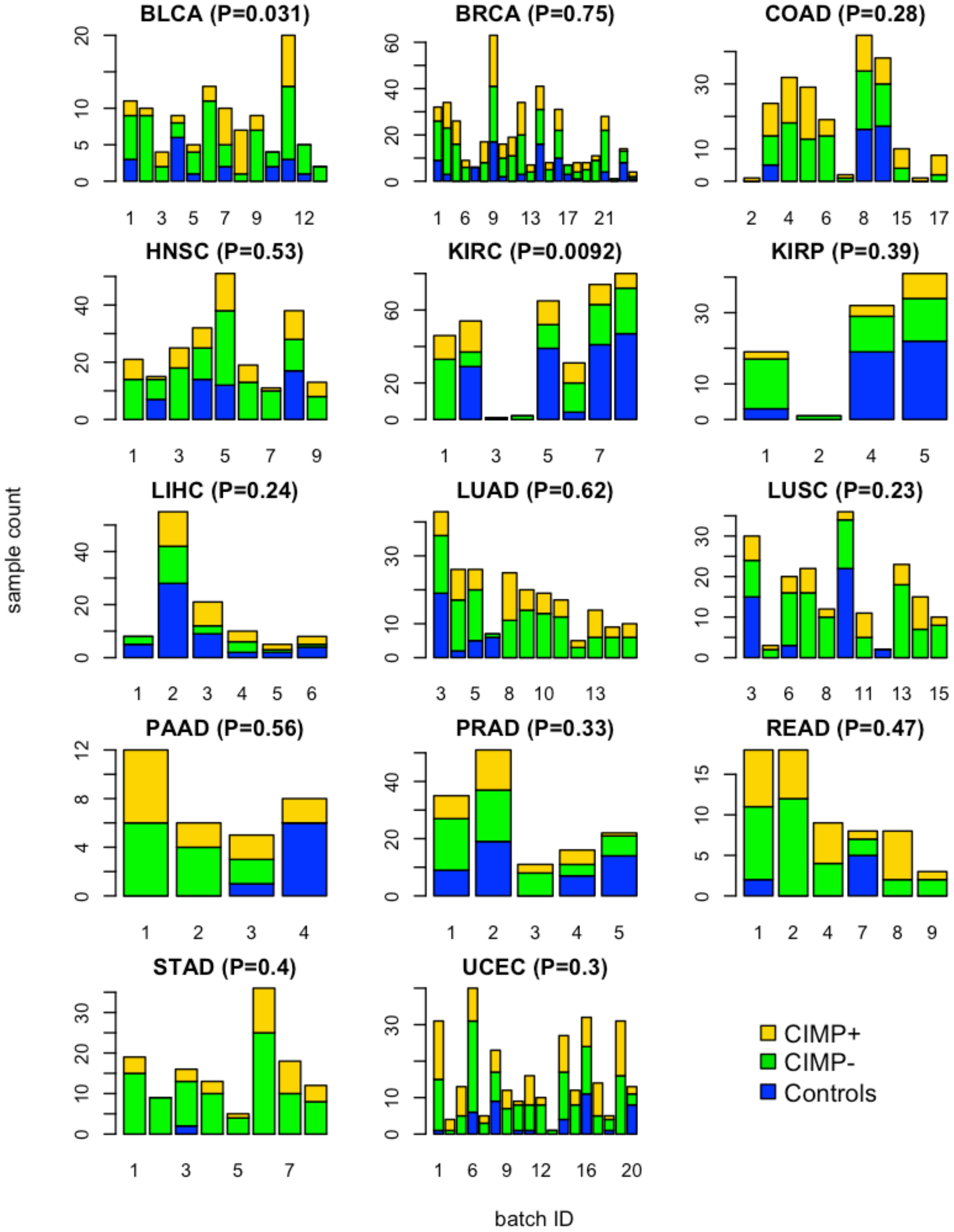

Supplement: Additional file 1: — Supplemental figures S1 to S12. [file 13072_2015_7_MOESM1_ESM.pdf]
