# Supplementary material for: Pan-cancer stratification of solid human epithelial tumors and cancer cell lines reveals commonalities and tissue-specific features of the CpG island methylator phenotype
Source: Epigenetics Chromatin. 2015 Apr 17;8:14. doi: 10.1186/s13072-015-0007-7 (PMC4424513; doi:10.1186/s13072-015-0007-7)
Supplement: Additional file 2: — Supplemental methods. [file 13072_2015_7_MOESM2_ESM.docx]

**Pan-cancer stratification of solid human epithelial tumors and cancer cell lines reveals commonalities and tissue-specific features of the CpG island methylator phenotype**

### Francisco Sánchez-Vega, Valer Gotea, Gennady Margolin, Laura Elnitski^§^

Translational and Functional Genomics Branch, National Human Genome Research Institute, National Institutes of Health, Bethesda, Maryland, United States of America.

^§^Corresponding author

Email addresses:

FSV: [sanchezf@cbio.mskcc.org](mailto:francisco.sanchezvega@nih.gov)

VG: [vgotea@nih.gov](mailto:vgotea@nih.gov)

GM: [Gennady.Margolin@nih.gov](mailto:Gennady.Margolin@nih.gov)

LE: [elnitski@mail.nih.gov](mailto:elnitski@mail.nih.gov)

# Supplemental Methods

**SM1 - Visualization of methylation patterns using probe density plots**

**SM2 - Algorithms**

**SM3 - Robustness of the CIMP+/- labels over different choices of probe selection thresholds and comparison with variance-guided feature selection**

**SM4 - LOTO cross-validation for assessment of sample classification accuracy**

**SM5 - Technical biases associated with Illumina probe types**

**SM6 - Impact of batch effects upon sample classification**

**SM1 - Visualization of methylation patterns using probe density plots**

The plots showing density of probes presented in Figs. S1 and S7 were drawn as contour plots using the output of the ‘kde2d’ function from the ‘MASS’ package in R. This function implements a two-dimensional kernel density estimator with a bivariate normal kernel that is evaluated on a square grid. We used the default normal reference bandwidth. Colors were normalized individually for each density plot, so that the maximum density value was shown in dark red and the minimum density value was shown in dark blue, with 256 equally spaced color levels representing density values between the two extremes.

**SM2. Algorithms**

*Sample classification algorithm*

Tumor samples for each individual cancer type were classified as CIMP+, CIMP- and CIMPi following a two-step process:

1. Feature selection: a set of differentially methylated probes was selected based on two requirements: (1) the probe must be located in a CpG island and (2) the mean level of methylation at the probe must be below 5% when computed across all control samples and above 25% when computed across all tumor samples.
2. Sample classification: each individual tumor sample was labeled as CIMP+, CIMP- or CIMPi based on its average level of methylation across the set of selected differentially methylated probes. Labels were assigned using k-means clustering with k=3 classes on the vector of mean values with length equal to the number of samples. The centroids for each of these three classes were initialized as the first, second and third quartiles of the distribution of mean sample methylation values (where the mean methylation value for each sample is computed over the corresponding differentially methylated probes). Of note, k-means classification using the vector of methylation values at differentially methylated sites (instead of their mean) to represent each sample yielded approximately equivalent results.

*Probe clustering algorithm*

As was the case for the sample classification algorithm, probe clustering is performed in two separate steps:

1. Ternary indicator computations: A ternary indicator variable is computed for each pair of probe and cancer type. This variable is equal to 1 if the probe exhibits higher levels of methylation in CIMP+ samples than in CIMP- samples for that cancer type, and it is equal to -1 if it exhibits lower levels of methylation in CIMP+ samples than in CIMP- samples. The variable takes value 0 if no differential methylation is observed. We consider that a given probe is differentially methylated in a given cancer type if (1) there is a statistically significant difference between the methylation values associated to CIMP+ and CIMP- samples based on a one-sided Wilcoxon rank-sum test (we required FDR<0.05 for multiple hypothesis correction across all probes) and (2) both the mean and median differences in methylation levels have absolute values above 10% (this second requirement was imposed in order to enforce a non-negligible magnitude of effect).
2. Sequential probe grouping: After all the indicator variables have been computed, we sort the probes according to their genomic locations. We apply a sequential algorithm that visits every single probe and groups together probes that satisfy the following conditions: (1) probes must be sequentially contiguous in the array, located within the same chromosome and located within a distance of 2 Kb and (2) in order for two probes to be assigned to the same cluster, we require their two associated vectors of differential methylation indicator variables to coincide in at least 10 out of the 12 cancer types.

**SM3 - Robustness of the CIMP+/- labels over different choices of probe selection thresholds and comparison with variance-guided feature selection**

We introduced the CIMPi (CIMP intermediate) class in our analysis in order to ensure that the tumors that we labeled as CIMP+ and CIMP- were sufficiently different by filtering out potentially borderline samples with intermediate levels of average methylation. Here we show that, after CIMPi samples are removed, the remaining CIMP+/- labels are very robust to different choices of probe selection parameters and also remain practically unchanged when an altogether different feature selection criterion is used.

First, we evaluated sensitivity of our classification results to different choices of α_C_ and α_T_ (Table S6). For this, we considered a set of discrete values that ranged between 0.01 and 0.15 for α_C_, and between 0.15 and 0.35 for α_T_. We reran our feature selection and sample classification algorithms for all pairwise combinations of these parameter choices and we counted the total number of CIMP+ and CIMP- samples that switched categories with respect to the original classification learned in our study, which had been obtained using α_C_ =0.05 and α_T_ =0.25. In the worst case combination, only 21 of 2,542 (0.8%) tumor samples having CIMP+/- were assigned switched labels. Therefore, we conclude that our CIMP+ and CIMP- labels are robust over a wide range of α_C_ and α_T_ parameter choices.

Second, we considered an alternative approach to feature selection which consists in selecting the 1,000 probes with the highest level of variance across samples (this is the same kind of variance selection approach used, for example, to define DNA methylation clusters in the TCGA marker paper for colorectal cancer [26]). As before, we reran our classification algorithm for each individual cancer type and, after discarding CIMPi samples, we counted the number of samples that were labeled as CIMP+ in our original classification and got CIMP- labels in the new classification (or viceversa). The results are shown in Table S7. At the pan-cancer level, only 21 of 2,542 (i.e., less than 1%) tumors switched labels. At the levels of invidual cancer types, the worst case corresponds to the LIHC cancer type, where 11 of 90 (i.e., 12.2%) samples switched labels. This results indicate that our CIMP+/- labels remain robust across different approaches to feature selection.

On a side note, we remark that CIMPi labels are much less robust to different strategies for probe selection (Tables S6 and S7). A non-negligible number of samples that are labeled as CIMPi (mainly those exhibiting borderline levels of average methylation) can be relabeled as CIMP+ or CIMP- when different choices of parameters are made, or when the variance selection approach is used. By contrast, some CIMP+/- samples may be relabeled as CIMPi based on different probe selection strategies, but it is extremely unlikely that a CIMP+ will become CIMP-, or viceversa. As we had already explained, this was one of the reasons why CIMPi samples were excluded from many parts of our analysis.

**SM4 - LOTO cross-validation for assessment of sample classification accuracy**

We did a leave-one-type-out (LOTO) analysis of classification accuracy for the panel of 89 pan-cancer markers shown in Table S1. This panel was selected as the set of probes that appears in the differentially methylated sets for at least 6 out of 14 different cancer types. We used the original set of CIMP+ and CIMP- labels as the reference ground truth for each individual cancer type. For each fold of the LOTO analysis, we excluded data for a specific cancer type and we selected the set of differentially methylated probes that appear in the original differentially methylated sets for at least 6 of the 13 remaining cancer types. Using those probes, we classified the CIMP+ and CIMP- samples from the excluded cancer type, which are therefore treated as an independent test set for that specific fold. We did this for all the 14 cancer types and we computed LOTO classification accuracy as the fraction of CIMP+ and CIMP- samples for which the LOTO labels agreed with the original labels that had been learned by using the entire dataset (Table S2). For comparison purposes, in that table we also provided a ‘shared set’ (S) estimate of classification accuracy, which was computed by using the unique shared set of 89 pan-cancer differentially methylated probes shown in Table S1 (in this case, the same set was used for all cancer types).

In order to assess statistical significance of our results, we performed a random permutations experiment. For each cancer type, we selected a random set of probes containing the same number of elements as the LOTO set that had been selected from data for that specific cancer type (as described below). Using that set of random probes, we computed classification accuracy and Spearman correlation between average level of methylation computed over the set of random probes and average level of methylation computed over all variably methylated probes (same type of correlation as reported in Fig. 1B). We run 10,000 replicates of the random probe selection process and we obtained empirical p-values as the percentage of replicates where the classification accuracy was equal to or higher than the one observed using the original LOTO set of selected probes (and similarly for correlations). Furthermore, we performed this experiment using two different strategies for random probe selection. First, we allowed probes to be randomly selected from anywhere in the Illumina array (results labeled as ‘AllP’ in Table S2). Then, we restricted the random choices of probes to include only probes located within CGIs (results labeled as ‘InCGI’ in Table S2). P-values in the ‘AllP’ column are extremely significant, which means that it is very unlikely to get LOTO classification rates and correlation coefficients as high as those that we had observed by choosing probes at random among all probes in the array. Some of the p-values in the ‘InCGI’ category are not significant. This suggests that the levels of methylation at probes located within CGIs are very strongly correlated, so that many different choices of random differentially methylated probes can provide very good classification and correlation results, which is consistent with the conclusions from our correlation analysis of variably methylated *vs*. differentially methylated probes (Fig. 1B).

**SM5 - Technical biases associated with Illumina probe types**

The Illumina HumanMethylation 450K arrays contain a mixture of Infinium I and Infinium II probe design types. Infinium II assays have been shown to exhibit larger variance and to be less sensitive to extreme methylation values [58,59]. Since our approach to feature selection is based on the search for extreme differences in average methylation levels for any given probe across samples, the differences in probe design tend to favor the selection of Type I probes. This, by itself, is not necessarily a problem, particularly since Type I probes have been reported to provide more accurate and more reproducible measurements of beta-values than Type II probes [58,59]. However, it order to make a better use of the information contained in Type II probes, we decided to normalize them using the BMIQ approach, which rescales them on the basis of their Infinium I array counterparts. The impact of this normalization procedure upon the number of probes in the differentially methylated set associated to each cancer type is shown in Fig S11A. As expected, a non-negligible number of Type II probes that would otherwise be ignored by our feature selection algorithm are chosen after normalization, potentially improving the accuracy of our method by weighing-in a larger amount of biologically relevant information. Figure S11B also shows that mean levels of methylation averaged over the sets of Type I and Type II differentially methylated probes are very strongly correlated, which suggests that sample classification based on the isolated use of either type of probes would be very similar to sample classification based on the combined use of the two probe types.

**SM6 - Impact of batch effects upon sample classification**

We carried out an exploratory analysis to evaluate the impact of potential batch effects upon our sample classification into the CIMP+ and CIMP- categories. Figure S12 shows the distribution of class labels assigned to samples as a function of their batch ID. We evaluated statistical association between the class label and the batch ID variables. This association was not statistically significant for 12 out of the 14 types in our analysis, with the only exceptions of BLCA and KIRC (with unadjusted p-values equal to 0.03 and 0.01 respectively). Reported p-values were computed using Fisher’s exact test. In the KIRC case, the significant association between class and batch variable is likely due to the fact that a couple of batch IDs contained very few instances of either class. In the BLCA case, several of the batches appear to be enriched for a certain choice of class label, although both CIMP+ and CIMP- samples are observed across most batch IDs. In general terms, these results show a small magnitude of batch effects, which should therefore not have an important impact upon the conclusions derived from our pan-cancer CIMP analysis.
