# Supplementary material for: Pan-cancer stratification of solid human epithelial tumors and cancer cell lines reveals commonalities and tissue-specific features of the CpG island methylator phenotype
Source: Epigenetics Chromatin. 2015 Apr 17;8:14. doi: 10.1186/s13072-015-0007-7 (PMC4424513; doi:10.1186/s13072-015-0007-7)
Supplement: Additional file 5: — Supplemental tables S1 to S7. [file 13072_2015_7_MOESM5_ESM.docx]

**Pan-cancer stratification of solid human epithelial tumors and cancer cell lines reveals commonalities and tissue-specific features of the CpG island methylator phenotype**

### Francisco Sánchez-Vega, Valer Gotea, Gennady Margolin, Laura Elnitski^§^

Translational and Functional Genomics Branch, National Human Genome Research Institute, National Institutes of Health, Bethesda, Maryland, United States of America.

^§^Corresponding author

Email addresses:

FSV: [sanchezf@cbio.mskcc.org](mailto:francisco.sanchezvega@nih.gov)

VG: [vgotea@nih.gov](mailto:vgotea@nih.gov)

GM: [Gennady.Margolin@nih.gov](mailto:Gennady.Margolin@nih.gov)

LE: [elnitski@mail.nih.gov](mailto:elnitski@mail.nih.gov)

# Supplemental Tables

**Table S1 - Panel of 89 pan-cancer markers to distinguish CIMP+ from CIMP- samples.**

**Table S2 - Results from LOTO analysis for the panel of 89 pan-cancer CIMP markers.**

**Table S3 - List of ENCODE human cell lines used in our DNA methylation analysis.**

**Table S4 - Cluster sizes and number of associated cancer types for differentially methylated neighborhoods.**

**Table S5 - Gene expression data files and sample sizes.**

**Table S6 - Robustness of CIMP+/- labels over different choices of probe selection thresholds.**

**Table S7 - Comparison of CIMP status labels with variance-guided probe selection method.**

## Table S1 - Panel of 89 pan-cancer markers to distinguish CIMP+ from CIMP- samples.

## Set of 89 probes that are proposed in the manuscript as a pan-cancer panel of discriminative sites to separate CIMP+ from CIMP- samples. This panel was defined as the set of differentially methylated sites that had been selected in at least 6 different cancer types. MAPINF and CpG Island coordinates correspond to hg19. ‘Location Category’ is shown as defined by Illumina.

| **IlmnID** | **CH** | **MAPINFO** | **RefGene Name** | **Location Category** | **CpG Island Coordinates** |
| --- | --- | --- | --- | --- | --- |
| cg00002719 | 1 | 169396706 | C1orf114 | TSS200 | chr1:169396621-169396869 |
| cg03257575 | 1 | 46951318 | <NA> | <NA> | chr1:46951168-46951792 |
| cg04123776 | 1 | 170630602 | <NA> | <NA> | chr1:170630456-170630851 |
| cg05377226 | 1 | 171810910 | DNM3 | 1stExon | chr1:171810467-171811325 |
| cg07790615 | 1 | 237205174 | RYR2 | TSS1500 | chr1:237205126-237206644 |
| cg10249375 | 1 | 63795934 | <NA> | <NA> | chr1:63795363-63796140 |
| cg10512745 | 1 | 50884480 | DMRTA2 | Body | chr1:50884228-50891471 |
| cg13939859 | 1 | 91183519 | BARHL2 | TSS1500 | chr1:91183240-91184540 |
| cg13958426 | 1 | 169396637 | C1orf114 | 1stExon;5'UTR | chr1:169396621-169396869 |
| cg14712186 | 1 | 91184413 | <NA> | <NA> | chr1:91183240-91184540 |
| cg15467646 | 1 | 22141014 | LDLRAD2 | Body | chr1:22140891-22141407 |
| cg15576900 | 1 | 44883697 | RNF220 | Body | chr1:44883136-44884272 |
| cg19807257 | 1 | 46951354 | <NA> | <NA> | chr1:46951168-46951792 |
| cg01419831 | 2 | 162283705 | <NA> | <NA> | chr2:162283581-162284677 |
| cg01576677 | 2 | 45029908 | <NA> | <NA> | chr2:45029728-45030035 |
| cg06382344 | 2 | 162280519 | TBR1 | Body | chr2:162279835-162280709 |
| cg06530490 | 2 | 118981783 | <NA> | <NA> | chr2:118981769-118982466 |
| cg11014373 | 2 | 105479054 | <NA> | <NA> | chr2:105478600-105479188 |
| cg12974388 | 2 | 237072998 | <NA> | <NA> | chr2:237071794-237078762 |
| cg14168530 | 2 | 45155991 | <NA> | <NA> | chr2:45155195-45157049 |
| cg20072442 | 2 | 80530255 | CTNNA2;LRRTM1 | Body | chr2:80529677-80530846 |
| cg21185289 | 2 | 74743437 | TLX2 | 3'UTR | chr2:74740455-74743795 |
| cg24632241 | 2 | 80530431 | CTNNA2;LRRTM1 | Body | chr2:80529677-80530846 |
| cg15994026 | 4 | 15780306 | CD38 | Body | chr4:15779998-15780729 |
| cg02511231 | 5 | 170738274 | TLX3 | Body | chr5:170735169-170739863 |
| cg08862890 | 5 | 169064451 | DOCK2 | Body | chr5:169064270-169064702 |
| cg12483476 | 5 | 178421711 | GRM6 | 1stExon | chr5:178421225-178422337 |
| cg14743594 | 5 | 167956394 | FBLL1 | TSS200 | chr5:167956219-167957371 |
| cg16150752 | 5 | 16180072 | MARCH11 | TSS200 | chr5:16179064-16180420 |
| cg17712694 | 5 | 16180068 | MARCH11 | TSS200 | chr5:16179064-16180420 |
| cg06463958 | 6 | 166582393 | T | TSS1500 | chr6:166579973-166583423 |
| cg08382226 | 6 | 108440339 | <NA> | <NA> | chr6:108437998-108441208 |
| cg08548396 | 6 | 30095549 | <NA> | <NA> | chr6:30095173-30095610 |
| cg14186641 | 6 | 88876741 | CNR1 | TSS1500 | chr6:88875397-88877449 |
| cg20295992 | 6 | 108440280 | <NA> | <NA> | chr6:108437998-108441208 |
| cg21277995 | 6 | 393239 | IRF4 | Body | chr6:391188-393790 |
| cg23164203 | 6 | 28367475 | ZSCAN12 | Body;5'UTR;1stExon | chr6:28367124-28367651 |
| cg27200446 | 6 | 41606439 | MDFI | 5'UTR | chr6:41604583-41606663 |
| cg27577527 | 6 | 28367544 | ZSCAN12 | TSS200 | chr6:28367124-28367651 |
| cg00469207 | 7 | 152622455 | <NA> | <NA> | chr7:152622343-152623305 |
| cg02467990 | 7 | 49813102 | VWC2 | TSS200 | chr7:49813008-49815752 |
| cg04904331 | 7 | 49813033 | VWC2 | TSS1500 | chr7:49813008-49815752 |
| cg09493505 | 7 | 49813111 | VWC2 | TSS200 | chr7:49813008-49815752 |

| cg13314145 | 7 | 98246001 | NPTX2 | TSS1500 | chr7:98245805-98247759 |
| --- | --- | --- | --- | --- | --- |
| cg17969084 | 7 | 27191097 | <NA> | <NA> | chr7:27190274-27191115 |
| cg18206027 | 7 | 49813486 | VWC2 | 1stExon;5'UTR | chr7:49813008-49815752 |
| cg20183619 | 7 | 155241490 | <NA> | <NA> | chr7:155241323-155243757 |
| cg21038156 | 7 | 158936739 | VIPR2 | Body | chr7:158936507-158938492 |
| cg21929943 | 7 | 152622815 | <NA> | <NA> | chr7:152622343-152623305 |
| cg25884711 | 7 | 24323840 | NPY | 5'UTR;1stExon | chr7:24323558-24325080 |
| cg02919936 | 8 | 70982285 | PRDM14 | 5'UTR | chr8:70981873-70984888 |
| cg05166490 | 8 | 41754172 | ANK1 | 1stExon;5'UTR | chr8:41753340-41755250 |
| cg13912117 | 8 | 132054555 | <NA> | <NA> | chr8:132052203-132054749 |
| cg15836635 | 8 | 57025662 | MOS | 1stExon | chr8:57025546-57026543 |
| cg20079899 | 8 | 72755784 | MSC | Body | chr8:72755783-72756667 |
| cg20980783 | 8 | 67874178 | <NA> | <NA> | chr8:67873388-67875600 |
| cg10143067 | 10 | 118892423 | VAX1 | Body | chr10:118892161-118892639 |
| cg14038391 | 10 | 102900130 | <NA> | <NA> | chr10:102899822-102900263 |
| cg03401096 | 11 | 123301171 | <NA> | <NA> | chr11:123301049-123302149 |
| cg06825142 | 11 | 637170 | DRD4 | TSS200 | chr11:636906-640628 |
| cg11017065 | 11 | 128564874 | FLI1;FLI1 | Body;5'UTR | chr11:128562671-128565011 |
| cg12928379 | 11 | 637175 | DRD4 | TSS200 | chr11:636906-640628 |
| cg15778437 | 11 | 31839521 | PAX6 | TSS200 | chr11:31839363-31839813 |
| cg17872757 | 11 | 128564180 | FLI1;FLI1 | Body;5'UTR | chr11:128562671-128565011 |
| cg22868282 | 12 | 47224954 | <NA> | <NA> | chr12:47224912-47225664 |
| cg24292235 | 12 | 8171463 | <NA> | <NA> | chr12:8171336-8171790 |
| cg26682580 | 12 | 128752058 | TMEM132C | Body | chr12:128751041-128753151 |
| cg14123923 | 13 | 79176572 | POU4F1 | Body | chr13:79175610-79177985 |
| cg19497031 | 13 | 79176272 | POU4F1 | Body | chr13:79175610-79177985 |
| cg04281464 | 14 | 70014873 | <NA> | <NA> | chr14:70014543-70014993 |
| cg05302386 | 14 | 52734525 | PTGDR | 1stExon;5'UTR | chr14:52734207-52735486 |
| cg05917460 | 14 | 37126902 | PAX9 | 1stExon;5'UTR | chr14:37126786-37128274 |
| cg13592399 | 14 | 52535758 | NID2;NID2 | 5'UTR;1stExon | chr14:52534581-52536722 |
| cg11601252 | 15 | 68122139 | LBXCOR1 | Body | chr15:68115485-68122575 |
| cg14394692 | 15 | 30517589 | <NA> | <NA> | chr15:30515266-30518445 |
| cg03217795 | 16 | 23847556 | PRKCB | 1stExon;1stExon | chr16:23846941-23848102 |
| cg05374412 | 16 | 82660727 | CDH13 | 1stExon | chr16:82660651-82661813 |
| cg11561737 | 17 | 46796908 | <NA> | <NA> | chr17:46796234-46797292 |
| cg15044248 | 17 | 75369224 | SEPT9 | TSS200;5'UTR;Body | chr17:75368688-75370506 |
| cg16779463 | 17 | 75369055 | SEPT9 | TSS1500;5'UTR;Body | chr17:75368688-75370506 |
| cg24761507 | 17 | 35293930 | LHX1 | TSS1500 | chr17:35291899-35300875 |
| cg20872937 | 18 | 74961968 | GALR1 | TSS200 | chr18:74961556-74963822 |
| cg03735888 | 19 | 58951602 | ZNF132 | TSS200 | chr19:58951214-58952250 |
| cg11957331 | 19 | 42828156 | TMEM145 | Body | chr19:42827743-42829149 |
| cg12042659 | 19 | 58951599 | ZNF132 | TSS200 | chr19:58951214-58952250 |
| cg12074025 | 19 | 58238850 | ZNF671 | 1stExon | chr19:58238585-58239028 |
| cg25875213 | 19 | 38183055 | ZNF781;ZNF781 | 5'UTR;1stExon | chr19:38182793-38183327 |
| cg03011535 | 20 | 42544794 | TOX2 | 5'UTR;Body;1stExon | chr20:42543097-42545137 |
| cg06962944 | 20 | 42544728 | TOX2 | TSS200;Body | chr20:42543097-42545137 |

## Table S2 - Results from LOTO analysis for the panel of 89 pan-cancer CIMP markers.

## The first column shows the number of probes selected in each fold of the LOTO cross validation loop. Columns 2 to 5 show the results for our analysis of classification accuracy. ‘Accu’ represents classification accuracy for each specific LOTO fold, using the set of LOTO specific differentially methylated probes with cardinality shown in the first column. ‘Accu(S)’ represents classification accuracy computed using the shared, fixed set of 89 pan-cancer differentially methylated probes. Two p-values are provided: the p-value labeled as ‘AllP’ comes from simulations where differentially methylated probes were randomly selected among the set of all probes in the array, while the p-value labeled as ‘inCGI’ comes from simulations where random choices of differentially methylated probes were restricted to probes located in CGIs. Columns 6 to 9 show equivalent results for the analysis of Spearman correlations between differentially methylated probes and variably methylated probes.

|  | # LOTO probes | CIMP+ *vs*. CIMP- Classification | | | | Spearman correlation | | | |
| --- | --- | --- | --- | --- | --- | --- | --- | --- | --- |
|  |  | Accu LOTO | p-val (AllP) | p-val (inCGI) | Accu (S) | rho (LOTO) | p-val (AP) | p-val (inCGI) | rho (S) |
| BLCA | 74 | 0.983 | 0.000 | 0.001 | 0.983 | 0.846 | 0.000 | 0.889 | 0.837 |
| BRCA | 51 | 0.998 | 0.000 | 0.000 | 0.998 | 0.922 | 0.000 | 0.131 | 0.925 |
| COAD | 58 | 0.967 | 0.000 | 0.076 | 0.995 | 0.920 | 0.000 | 0.540 | 0.943 |
| HNSC | 51 | 0.996 | 0.000 | 0.000 | 0.996 | 0.833 | 0.000 | 0.735 | 0.862 |
| KIRC | 89 | 0.970 | 0.000 | 0.006 | 0.970 | 0.935 | 0.000 | 0.162 | 0.935 |
| KIRP | 88 | 0.716 | 0.520 | 0.988 | 0.716 | 0.886 | 0.000 | 0.207 | 0.888 |
| LIHC | 65 | 0.944 | 0.000 | 0.076 | 0.967 | 0.761 | 0.020 | 0.960 | 0.757 |
| LUAD | 45 | 0.985 | 0.000 | 0.006 | 0.989 | 0.879 | 0.000 | 0.591 | 0.890 |
| LUSC | 49 | 0.945 | 0.000 | 0.021 | 0.991 | 0.813 | 0.006 | 0.707 | 0.832 |
| PAAD | 60 | 1.000 | 0.000 | 0.066 | 1.000 | 0.939 | 0.000 | 0.131 | 0.939 |
| PRAD | 79 | 0.984 | 0.001 | 0.259 | 0.992 | 0.947 | 0.002 | 0.283 | 0.947 |
| READ | 60 | 1.000 | 0.000 | 0.000 | 1.000 | 0.806 | 0.003 | 0.630 | 0.793 |
| STAD | 75 | 0.909 | 0.000 | 0.934 | 0.958 | 0.791 | 0.053 | 1.000 | 0.814 |
| UCEC | 48 | 0.996 | 0.000 | 0.000 | 1.000 | 0.912 | 0.000 | 0.063 | 0.911 |
| Avg. | 63.71 | 0.957 | -- | -- | 0.968 | 0.871 | -- | -- | 0.877 |

## Table S3 – List of human cell lines from ENCODE used in our DNA methylation analysis.

## Human cell lines from ENCODE and corresponding annotations (from <http://genome.ucsc.edu/ENCODE/cellTypes.html>). Only cell lines annotated as having ‘cancer’ or ‘non-cancer’ karyotypes were used in our study.

| **Cell line** | **Description** | **Lineage** | **Tissue** | **Karyo** |
| --- | --- | --- | --- | --- |
| A549 | epithelial cell line derived from a lung carcinoma tissue | endoderm | epithelium | cancer |
| AG04449 | fetal buttock/thigh fibroblast | -- | skin | normal |
| AG04450 | fetal lung fibroblast | endoderm | lung | normal |
| AG09309 | adult toe fibroblast | -- | skin | -- |
| AG09319 | gum tissue fibroblasts | -- | gingival | normal |
| AG10803 | abdominal skin fibroblasts | -- | skin | -- |
| AoSMC | aortic smooth muscle cells | mesoderm | blood vessel | normal |
| BE2_C | neuroblastoma | ectoderm | brain | cancer |
| BJ | skin fibroblast | -- | skin | normal |
| Caco-2 | colorectal adenocarcinoma | endoderm | colon | cancer |
| CMK | acute megakaryocytic leukemia cells | mesoderm | blood | cancer |
| ECC-1 | epithelial cell line derived from an endometrium adenocarcinoma | endoderm | luminal epithelium | cancer |
| GM06990 | B-lymphocyte, lymphoblastoid | mesoderm | blood | -- |
| GM12878 | B-lymphocyte, lymphoblastoid | mesoderm | blood | normal |
| GM12891 | B-lymphocyte, lymphoblastoid | mesoderm | blood | -- |
| GM12892 | B-lymphocyte, lymphoblastoid | mesoderm | blood | -- |
| GM19239 | B-lymphocyte, lymphoblastoid | mesoderm | blood | -- |
| H1-hESC | embryonic stem cells | inner cell mass | embryonic stem cell | normal |
| HAEpiC | amniotic epithelial cells | -- | epithelium | normal |
| HCF | cardiac fibroblasts | mesoderm | heart | normal |
| HCM | cardiac myocytes | mesoderm | heart | normal |
| HCPEpiC | choroid plexus epithelial cells | ectoderm | epithelium | normal |
| HCT-116 | colorectal carcinoma | endoderm | colon | cancer |
| HEEpiC | esophageal epithelial cells | endoderm | epithelium | normal |
| HEK293 | embryonic kidney | mesoderm | kidney | -- |
| HeLa-S3 | cervical carcinoma | ectoderm | cervix | cancer |
| Hepatocytes | primary hepatocytes | endoderm | liver | normal |
| HepG2 | hepatocellular carcinoma | endoderm | liver | cancer |
| HIPEpiC | iris pigment epithelial cells | ectoderm | epithelium | normal |
| HL-60 | promyelocytic leukemia cells | mesoderm | blood | cancer |
| HMEC | mammary epithelial cells | ectoderm | breast | normal |
| HNPCEpiC | non-pigment ciliary epithelial cells | -- | epithelium | normal |
| HPAEpiC | pulmonary alveolar epithelial cells | -- | epithelium | normal |
| HRE | renal epithelial cells | mesoderm | epithelium | normal |
| HRPEpiC | retinal pigment epithelial cells | ectoderm | epithelium | normal |
| HUVEC | umbilical vein endothelial cells | mesoderm | blood vessel | normal |
| IMR90 | fetal lung fibroblasts | endoderm | lung | normal |
| Jurkat | T lymphoblastoid derived from an acute T cell leukemia | mesoderm | blood | cancer |
| K562 | leukemia | mesoderm | blood | cancer |
| LNCaP | prostate adenocarcinoma | endoderm | prostate | cancer |
| MCF10A-Er-Src | mammary gland, non-tumorigenic epithelial | ectoderm | breast | -- |
| MCF10A-Es-Tam | mammary gland, non-tumorigenic epithelial | ectoderm | breast | -- |
| MCF-7 | mammary gland, adenocarcinoma | ectoderm | breast | cancer |
| NB4 | acute promyelocytic leukemia cell line | mesoderm | blood | cancer |
| NH-A | astrocytes | ectoderm | brain | normal |
| NHBE | bronchial epithelial cells | endoderm | epithelium | normal |
| NHDF-neo | neonatal dermal fibroblasts | mesoderm | skin | normal |
| NT2-D1 | malignant pluripotent embryonal carcinoma | inner cell mass | testis | cancer |
| ovcar-3 | ovarian adenocarcinoma | mesoderm | ovary | cancer |
| PANC-1 | pancreatic carcinoma | endoderm | pancreas | cancer |
| PFSK-1 | neuroectodermal cell line derived from a cerebral brain tumor | ectoderm | brain | cancer |
| PrEC | prostate epithelial cell line | epithelial | prostate | normal |
| ProgFib | fibroblasts, Hutchinson-Gilford progeria syndrome | -- | skin | -- |
| RPTEC | renal proximal tubule epithelial cells | -- | epithelium | normal |
| SAEC | small airway epithelial cells | endoderm | epithelium | normal |
| SkMC | Skeletal Striated Muscle Cells | missing | muscle | normal |
| SK-N-MC | neuroepithelioma cell line derived from a metastatic supra-orbital human brain tumor | ectoderm | brain | cancer |
| SK-N-SH_RA | neuroblastoma | ectoderm | brain | cancer |
| SK-N-SH_S | human neuroblastoma | ectoderm | brain | cancer |
| T-47D | epithelial cell line derived from a mammary ductal carcinoma. | -- | breast | cancer |
| U87 | glioblastoma, astrocytoma | ectoderm | brain | cancer |

## Table S4 - Cluster sizes and number of associated cancer types for differentially methylated probe clusters.

## Number of regions (clusters of probes) selected using our probe clustering algorithm. Columns show the number of probes within each cluster. Rows show the number of different cancer types showing differential methylation at probes contained in the cluster

|  |  | Cluster size | | | | | | | | | | | | | | | Total |
| --- | --- | --- | --- | --- | --- | --- | --- | --- | --- | --- | --- | --- | --- | --- | --- | --- | --- |
|  |  | 1 | 2 | 3 | 4 | 5 | 6 | 7 | 8 | 9 | 10 | 11 | 12 | 13 | 14-18 | ≥19* |  |
| Number of cancer types | 1 | 22951 | 4360 | 1860 | 907 | 504 | 293 | 172 | 118 | 86 | 59 | 41 | 40 | 15 | 37 | 10 | 31453 |
|  | 2 | 16929 | 2734 | 1119 | 515 | 286 | 168 | 112 | 60 | 48 | 32 | 20 | 25 | 8 | 23 | 7 | 22086 |
|  | 3 | 11711 | 1786 | 762 | 338 | 189 | 108 | 76 | 46 | 45 | 29 | 15 | 9 | 8 | 14 | 8 | 15144 |
|  | 4 | 8752 | 1250 | 480 | 235 | 110 | 76 | 42 | 35 | 21 | 19 | 15 | 8 | 5 | 8 | 3 | 11059 |
|  | 5 | 5804 | 862 | 352 | 164 | 90 | 50 | 33 | 28 | 18 | 7 | 9 | 3 | 10 | 5 | 4 | 7439 |
|  | 6 | 3750 | 672 | 279 | 156 | 86 | 42 | 26 | 19 | 9 | 9 | 10 | 2 | 1 | 1 | 2 | 5064 |
|  | 7 | 2532 | 617 | 266 | 114 | 53 | 55 | 36 | 24 | 9 | 10 | 3 | 2 | 4 | 5 | 1 | 3731 |
|  | 8 | 1892 | 561 | 235 | 152 | 75 | 38 | 29 | 30 | 15 | 15 | 5 | 3 | 5 | 7 | 3 | 3065 |
|  | 9 | 1511 | 524 | 224 | 140 | 80 | 48 | 28 | 20 | 15 | 10 | 5 | 8 | 2 | 8 | 3 | 2626 |
|  | 10 | 1011 | 412 | 252 | 152 | 85 | 66 | 32 | 22 | 16 | 13 | 6 | 4 | 3 | 6 | 3 | 2083 |
|  | 11 | 617 | 286 | 168 | 98 | 67 | 47 | 32 | 15 | 11 | 10 | 4 | 4 | 3 | 8 | 4 | 1374 |
|  | 12 | 278 | 149 | 95 | 81 | 50 | 23 | 19 | 13 | 8 | 7 | 8 | 5 | 6 | 7 | 2 | 751 |
| Total | | 77738 | 14213 | 6092 | 3052 | 1675 | 1014 | 637 | 430 | 301 | 220 | 141 | 113 | 70 | 129 | 50 | 105875 |

## Table S5 - Gene expression data files and sample sizes.

## For each cancer type, we provide the directory name with the exact version of the data that we used in our analysis. We also list the number of samples with available gene expression data.

| Type | Gene expressiong directory | Number of samples |
| --- | --- | --- |
| BLCA | unc.edu_BLCA.IlluminaHiSeq_RNASeqV2.Level_3.1.10.0 | 230 |
| BRCA | unc.edu_BRCA.IlluminaHiSeq_RNASeqV2.Level_3.1.5.0 | 1106 |
| COAD | unc.edu_COAD.IlluminaHiSeq_RNASeqV2.Level_3.1.6.0; unc.edu_COAD.IlluminaGA_RNASeqV2.Level_3.1.0.0 | 445 |
| HNSC | unc.edu_HNSC.IlluminaHiSeq_RNASeqV2.Level_3.1.5.0 | 467 |
| KIRC | unc.edu_KIRC.IlluminaHiSeq_RNASeqV2.Level_3.1.3.0 | 579 |
| KIRP | unc.edu_KIRP.IlluminaHiSeq_RNASeqV2.Level_3.1.10.0 | 191 |
| LIHC | unc.edu_LIHC.IlluminaHiSeq_RNASeqV2.Level_3.1.7.0 | 197 |
| LUAD | unc.edu_LUAD.IlluminaHiSeq_RNASeqV2.Level_3.1.12.0 | 548 |
| LUSC | unc.edu_LUSC.IlluminaHiSeq_RNASeqV2.Level_3.1.7.0 | 532 |
| PAAD | unc.edu_PAAD.IlluminaHiSeq_RNASeqV2.Level_3.1.3.0 | 58 |
| PRAD | unc.edu_PRAD.IlluminaHiSeq_RNASeqV2.Level_3.1.6.0 | 301 |
| READ | unc.edu_READ.IlluminaHiSeq_RNASeqV2.Level_3.1.6.0; unc.edu_READ.IlluminaGA_RNASeqV2.Level_3.1.0.0 | 162 |
| UCEC | unc.edu_UCEC.IlluminaHiSeq_RNASeqV2.Level_3.1.5.0; unc.edu_UCEC.IlluminaGA_RNASeqV2.Level_3.1.0.0 | 517 |

## Table S6 - Robustness of CIMP+/- labels over different choices of probe selection thresholds.

## The top part of the table shows the number of samples originally labeled as CIMP+ that were labeled as CIMP- when using the new probe selection threshold (or viceversa). A total of 2,542 samples had been labeled as either CIMP+ or CIMP- in our work. The choice α_C_=0.025 and α_T_ =0.35 led to no discriminative probes being selected in BLCA, BRCA, KIRP and PRAD, so samples from those types were excluded from the counts for that specific choice of thresholds. The bottom part of the table shows the percentage of label agreement for different choices of parameterization, computed using all the tumor samples (including CIMPi).

| Number of CIMP+/- samples with reversed labels | | Choice of **α**_T_ | | | | |
| --- | --- | --- | --- | --- | --- | --- |
|  |  | 0.15 | 0.20 | 0.25 | 0.30 | 0.35 |
| Choice of **α**_C_ | 0.025 | 0 | 0 | 3 | 21 | 17 |
|  | 0.05 | 0 | 0 | 0 | 0 | 0 |
|  | 0.10 | 0 | 0 | 0 | 0 | 0 |
|  | 0.15 | 0 | 0 | 0 | 0 | 0 |
| Percentage of matching tumor labels (including CIMPi) | | Choice of **α**_T_ | | | | |
|  |  | 0.15 | 0.20 | 0.25 | 0.30 | 0.35 |
| Choice of **α**_C_ | 0.025 | 79.8% | 85.9% | 84.3% | 77.7% | 75.7% |
|  | 0.05 | 85.9% | 92.3% | 100.0% | 89.7% | 86.0% |
|  | 0.10 | 86.7% | 91.2% | 92.7% | 89.6% | 85.5% |
|  | 0.15 | 86.2% | 90.1% | 89.7% | 88.1% | 83.9% |

## Table S7 - Comparison of CIMP status labels with variance-guided probe selection method.

## The first row shows the number of samples originally labeled as CIMP+ using our original probe selection approach that were labeled as CIMP- when using the variance approach for probe selection (or viceversa). The number of samples labeled as CIMP+ or CIMP- is also provided for each individual cancer type (e.g., in BLCA, 0/117 means that there were 0 samples which switched CIMP+/- labels out of 117 samples originally labeled as either CIMP+ or CIMP-). The second row shows the percentage of label agreement between the two probe selection methods, computed using all the tumor samples (including CIMPi).

| BLCA | BRCA | COAD | HNSC | KIRC | KIRP | LIHC | LUAD | LUSC | PAAD | PRAD | READ | STAD | UCEC |
| --- | --- | --- | --- | --- | --- | --- | --- | --- | --- | --- | --- | --- | --- |
| 0/117 | 0/432 | 0/182 | 1/240 | 6/202 | 1/88 | 11/90 | 0/268 | 1/217 | 0/32 | 0/126 | 0/57 | 1/165 | 0/268 |
| 77.1% | 80.5% | 88.0% | 75.1% | 62.8% | 69.4% | 39.7% | 77.1% | 79.7% | 76.9% | 89.1% | 75.0% | 88.5% | 86.5% |
